# Organ-Specific Gene Expression Control Using DNA Origami-Based Nanodevices

**DOI:** 10.1021/acs.nanolett.4c02104

**Published:** 2024-06-26

**Authors:** Yuxiang Liu, Ruixuan Wang, Qimingxing Chen, Yan Chang, Qi Chen, Kodai Fukumoto, Bingxun Wang, Jianchen Yu, Changfeng Luo, Jiayuan Ma, Xiaoxia Chen, Yuko Murayama, Kenichi Umeda, Noriyuki Kodera, Yoshie Harada, Shun-ichi Sekine, Jianfeng Li, Hisashi Tadakuma

**Affiliations:** † School of Life Science and Technology, ShanghaiTech University, Shanghai 201210 People’s Republic of China; ‡ Institute for Protein Research, Osaka University, Osaka 565-0871, Japan; § RIKEN Center for Biosystems Dynamics Research, Yokohama 230-0045, Japan; ∥ Zhejiang Provincial Key Laboratory of Pancreatic Disease Hangzhou, Zhejiang University School of Medicine First Affiliated Hospital, Zhejiang 310009, People’s Republic of China; ⊥ Nano Life Science Institute (WPI-NanoLSI), Kanazawa University, Kakuma-machi, Kanazawa 920-1192, Japan; # Gene Editing Center, School of Life Science and Technology, ShanghaiTech University, Shanghai 201210, People’s Republic of China

**Keywords:** DNA origami, LNP, Cryo-EM, gene expression

## Abstract

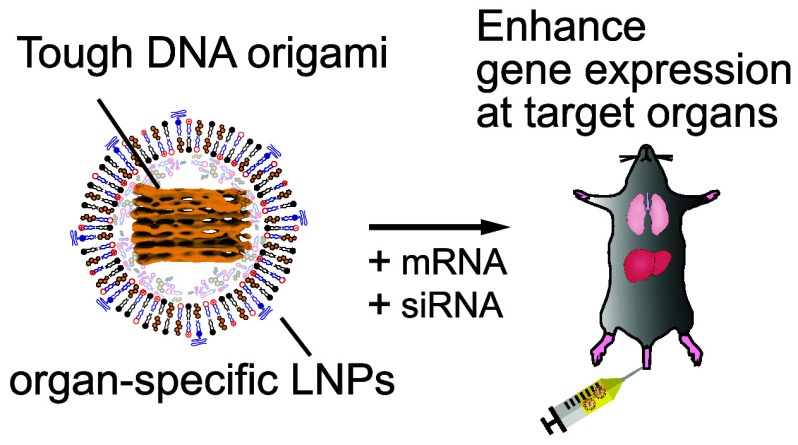

Nanodevices that function in specific organs or cells are one of the ultimate goals of synthetic biology. The recent progress in DNA nanotechnology such as DNA origami has allowed us to construct nanodevices to deliver a payload (e.g., drug) to the tumor. However, delivery to specific organs remains difficult due to the fragility of the DNA nanostructure and the low targeting capability of the DNA nanostructure. Here, we constructed tough DNA origami that allowed us to encapsulate the DNA origami into lipid-based nanoparticles (LNPs) under harsh conditions (low pH), harnessing organ-specific delivery of the gene of interest (GOI). We found that DNA origami-encapsulated LNPs can increase the functionality of payload GOIs (mRNA and siRNA) inside mouse organs through the contribution from different LNP structures revealed by cryogenic electron microscope (Cryo-EM). These data should be the basis for future organ-specific gene expression control using DNA origami nanodevices.

DNA origami is a versatile method used to construct custom two- and three-dimensional structures.^1−7^ Due to its predictable and programmable character, DNA origami allows precise control of the number, density, and layout of integrated molecules, making DNA origami an ideal platform for the delivery of molecules to cells. Recent advances have achieved highly efficient tumor treatment in mice, where tube-like or cage-like DNA origami has been used to deliver payloads to tumors.^8−10^ However, except for tumor delivery, *in vivo* delivery of DNA origami to the target site still has challenges. In particular, the clearance issue, i.e., DNA origami is easily cleared by the liver/kidney and externally discharged from urine, is the major challenge.^11^ Therefore, a versatile method to overcome these clearance issues has been demanded.

COVID-19 mRNA vaccines have demonstrated the power of lipid nanoparticle (LNP) technology. Fragile mRNAs are encapsulated by lipid-based nanoparticles and can be maintained in the body for a long period of period. Conventional LNPs comprise four components: ionizable cationic lipids, amphipathic phospholipids, cholesterol, and poly(ethylene glycol) (PEG) lipids.^12^ Recently, by introducing a fifth element, a selective organ-targeting (SORT) molecule, researchers succeeded in rationally designing nanoparticles that selectively target specific tissues.^13−16^ In this approach, the optimized internal charge of LNPs can alter the LNPs’ *in vivo* organ-targeting properties, enabling the delivery of mRNA to nonliver tissues. Therefore, this SORT technology might help overcome the clearance issue of DNA origami. However, to the best of our knowledge, the application of SORT technology to DNA origami is limited. Probably, the breakdown of DNA origami by harsh encapsulation conditions (e.g., use of low pH to encapsulate payload) has hindered its application.

Here, we made DNA origami tough by introducing internal UV cross-linking through a thymine dimer. Our data showed that UV cross-linked tough DNA origami is not toxic to cells, while it is important to maintain DNA origami intact after LNP encapsulation. LNP-encapsulated origami can target specific organs as designed, showing that SORT technology can be applied to DNA origami even in the unique surface charge situation of DNA origami.^17−19^ Moreover, we found that DNA origami can enhance the function of a gene of interest (GOI, e.g., mRNA and siRNA) *in vivo* in specific organs (lung and liver). These data showed that LNP technology helps DNA origami to overcome the clearance issue, and *vice versa*, DNA origami helps GOI encapsulated in LNPs to function *in vivo*. The combination of UV cross-linked tough DNA origami technology and LNP technology is thus straightforward to realize nanodevice functioning *in vivo*.

We used 42 helix-bundle (42hb) as a model DNA origami, which has a rod shape and can be easily used as a breadboard to integrate biomolecules (Scheme 1, Figure 1, Supplementary Figure 1, and Supplementary Table 1). Before testing the LNP encapsulation of 42hb, we first focused on making DNA origami tough, as encapsulation conditions (e.g., low pH) would affect the DNA origami structure. We reasoned that UV cross-linking of the DNA origami structure through the introduction of covalent bonds (thymine dimer) at user-defined sites is a suitable method to make DNA origami tough because UV cross-linking makes DNA origami highly resistant to nucleases and temperature while retaining their functionality (e.g., hybridization capability).^20,21^ Moreover, the addition of thymine nucleotides into staples is much more cost-effective than chemical modification of staple strands (e.g., phosphorothioates, PSs), and the photocontrol approach has better reproducibility than the solution-based modification approach (e.g., ligation).

**Scheme 1:**
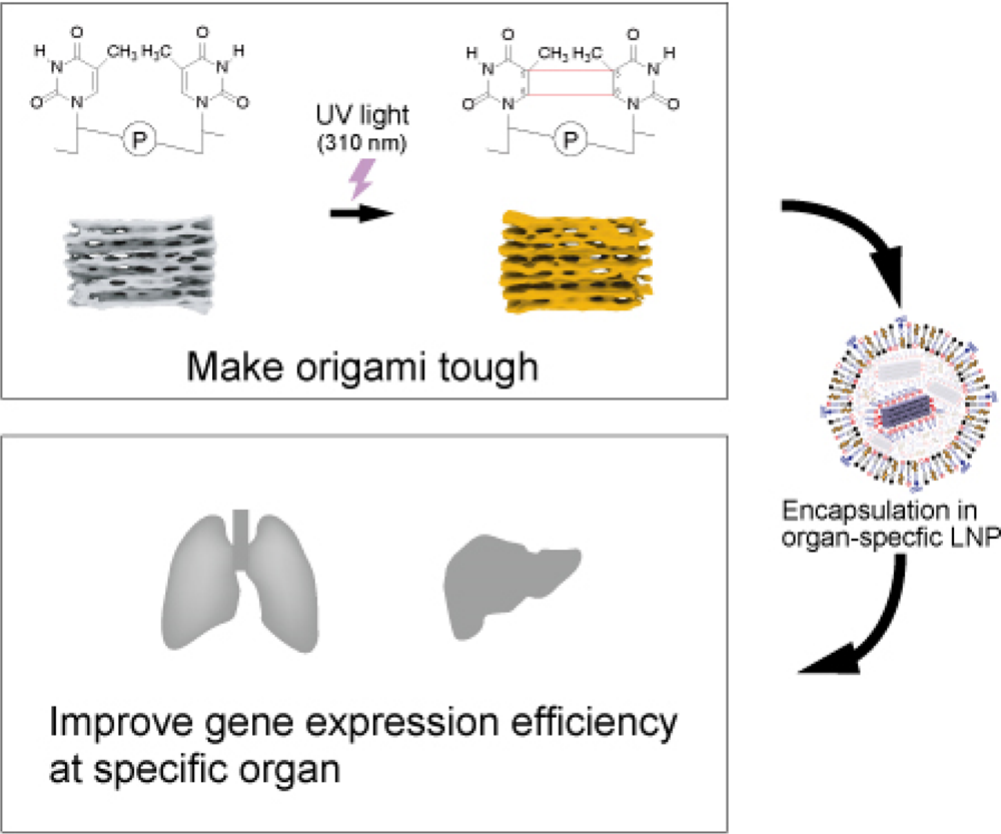
Schematic Illustration of Organ-Specific Gene Expression Control by DNA Origami-Based Nanodevices

**Figure 1:**
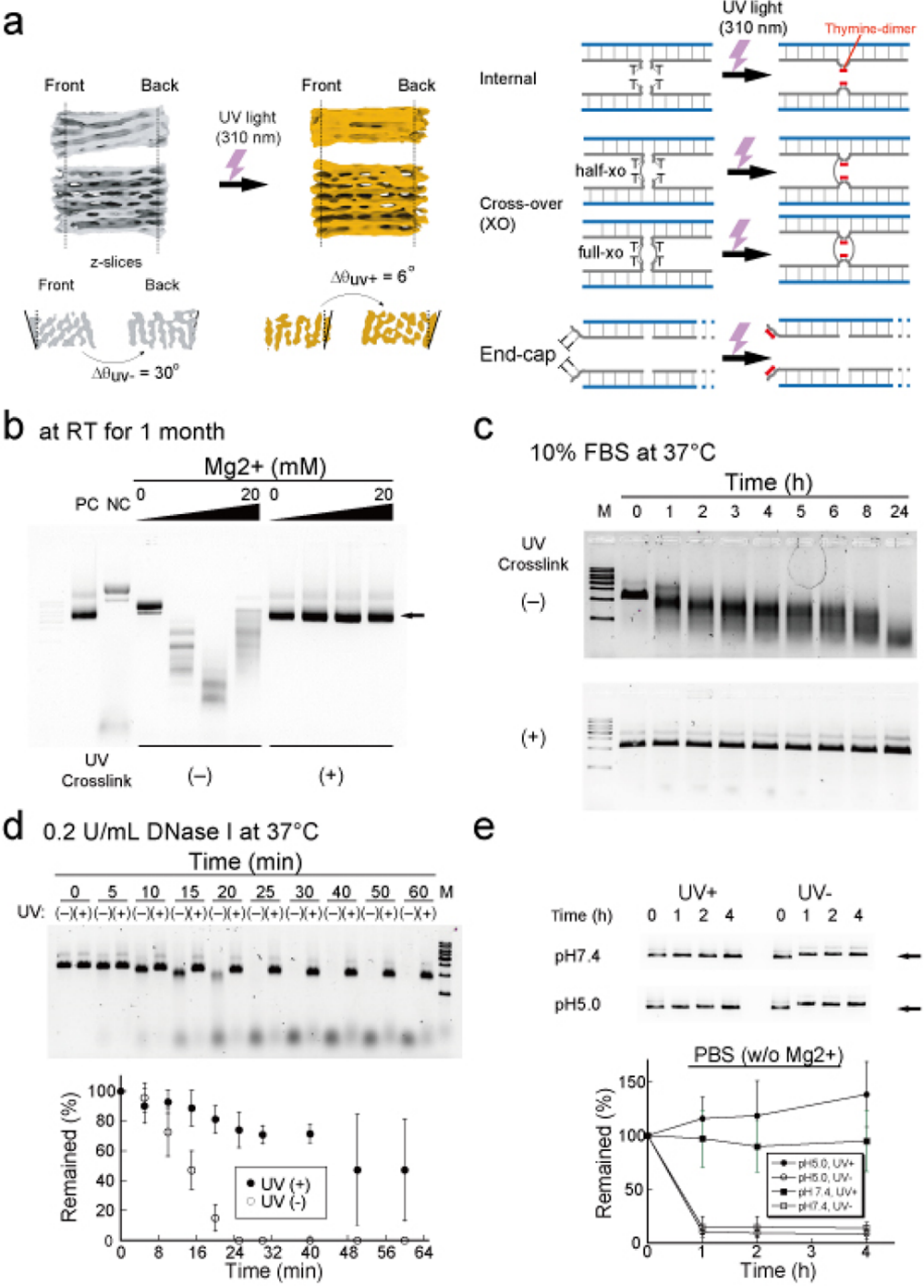
UV cross-linking improves the toughness of DNA origami. (a) (Left) Cryo-EM maps of DNA origami (42hb) before (left, gray) and after (right, yellow) UV irradiation (see also Supplementary Figure 2). The dashed lines indicate the front and back sides of the origami, showing torsional relief following UV treatment. (Right) UV irradiation induces thymine dimers at the internal, crossover, and end parts of the DNA origami. (b) Agarose gel electrophoresis of 42hb stored at 1 μM at room temperature for 1 month under low Mg^2+^ buffer (0, 5, 10, or 20 mM). PC: positive control freshly prepared 42hb. NC: negative control, unfolded mixture of scaffold and staples of 42hb. The arrow indicates the position of the intact 42hb structure. (c) Agarose gel electrophoresis of 42hb incubated in PBS with 10% FBS (without supplement Mg^2+^ ion) at 37 °C up to 24 h. (d) Agarose gel electrophoresis of 42hb digested by 0.2 U/mL DNase I at 37 °C up to 1 h. Error bars indicate the standard deviation of three independent experiments. (e) Agarose gel electrophoresis of 42hb incubated in PBS (pH 7.4 or 5.0, without supplement Mg^2+^ ion) at 37 °C for up to 4 h. The arrow indicates the position of the intact 42hb structure. Error bars indicate standard deviation of three independent experiments.

After folding 42hb, we introduced the thymine dimer by irradiating it with UV light (310 nm) for 1 h. We chose 310 nm based on previous reports^20,21^ and our experience^22^ to avoid DNA damage while achieving high cross-link density through the thymine dimer. As we added additional thymine both at all strand terminals and at all strand crossover points (Figure 1a right), internal covalent bonding must be introduced between staple–staple and helix-bundles by UV irradiation. Furthermore, thymines at the terminus of the structure (we used 10 thymines) would form thymine capping (end-cross-linked DNA) through UV irradiation.^21^

To confirm the effect of UV irradiation, we used a cryo-electron microscope (Cryo-EM) and analyzed the structure of 42hb before and after UV irradiation (Figure 1a, Supplementary Figure 2). As reported,^20^ we observed the preservation of the global shape while also observing the torsional relief (twist deformation) between the front and back sides (from 30° to 6° upon UV irradiation), suggesting that the structure showed the signature of UV irradiation. Upon confirming the effect of UV irradiation on 42hb, we examined its stability. Usually, DNA origami requires a substantial magnesium ion (Mg^2+^) concentration to maintain the structure (counterions, i.e., magnesium ions, suppress repulsive force caused by the negatively charged DNA strand). Thus, removing Mg^2+^ breaks down the DNA origami structure. To assess the effect of UV cross-linking on DNA origami structure, we incubated 42hb with different Mg^2+^ concentrations (Figure 1b). We found that UV-cross-linked DNA origami (hereafter tough DNA origami) can survive 1 month under low Mg^2+^ conditions, even in pure water (Milli-Q water), whereas DNA origami structures without UV cross-linking were easily unfolded or even degraded (probably caused by DNase contamination). Under cell culture conditions (10% fetal bovine serum, FBS, supplemented with PBS) at 37 °C, tough DNA origami survives for more than 24 h, whereas DNA origami structures without UV cross-linking broke down within 1 h (Figure 1c). Similarly, tough DNA origami remained against DNaseI (Figure 1d), low pH (acid condition, pH5, Figure 1e), and high temperature (Supplementary Figure 3), showing that UV cross-linking makes DNA origami tougher against harsh environmental conditions.

In natural cells, thymine dimers affect cell activities and are, therefore, quickly removed from the genome. To evaluate the effect of thymine-dimer incorporation on DNA origami, we performed cell uptake and cell viability tests (Figure 2 and Supplementary Figure 4). For cell uptake, we simply applied Cy5 fluorescent dye-labeled 42hb to the cell culture medium (final concentration of 10 nM). We evaluated three samples: UV cross-linked (UV+, tough origami), without UV cross-linked (UV−) and Cy5 oligo. Upon application to the cell medium, all three samples were spontaneously taken up similar to reported uptake for the UV– sample.^23,24^ The Cy5 oligo was taken up the fastest among the three conditions for all cell lines we tested (HEK293T, a human kidney cell line; H1299, a human lung carcinoma cell; HeLa, a human cervical cancer cell; L929, a mouse fibroblast cell; K562, a human leukemia cell). UV– samples were the second fastest sample. And UV+ was the slowest. The faster cell uptake of UV– samples compared with UV+ (tough) samples presumably reflects the breakdown tendency of UV– samples in the cell culture medium (Figure 2c), and some unfolded Cy5 staples were taken up earlier than the remaining body part of DNA origami. We note that the observed cell uptake was highly assisted by the Cy5 fluorescent dye (Supplementary Figure 5). The cell uptake speed was highly dependent on the cell line. The uptake of K562 cells (floating cells) was much faster, while HeLa cell uptake was slower and limited. Confocal images showed the Cy5-origami signal in the nucleus after 24 h, suggesting the endosomal escape of taken up DNA origami (Figure 2c). To elucidate the uptake mechanism, we used 4 inhibitors: Poly I for scavenger receptors, methyl-beta-cyclodextrin for caveolin-dependent endocytosis, cytochalasin D for nonreceptor-mediated endocytosis, and sucrose for clathrin-dependent endocytosis (Figure 2d). The major cell uptake pathway depends on the cell line. The majority of the cells we tested (HEK293T, H1299, and HeLa) depended on the scavenger receptor (inhibited by polyI), as reported.^23,24^ Minor cells depended on nonreceptor-mediated endocytosis (inhibited by cytochalasin D, L929 cell) or caveolin-dependent endocytosis (inhibited by methyl-beta-cyclodextrin, K562 cell) and presumably depended on the receptor expression patterns. Overall, inhibitors showed effects on thymine-dimer-introduced UV (+, tough) DNA origami similar to that of UV (−), suggesting that thymine-dimer introduction does not significantly affect the DNA origami behavior at the cell uptake stage. Moreover, a cell viability test using CCK8, a method to measure cellular dehydrogenase activity, showed that UV (+) tough DNA origami did not induce significant damage to cells: rather a slight increase in cellular activity was observed (Supplementary Figure 4). These data showed that UV-cross-linked tough DNA origami was not toxic to cellular activities at our currently applied dose.

**Figure 2:**
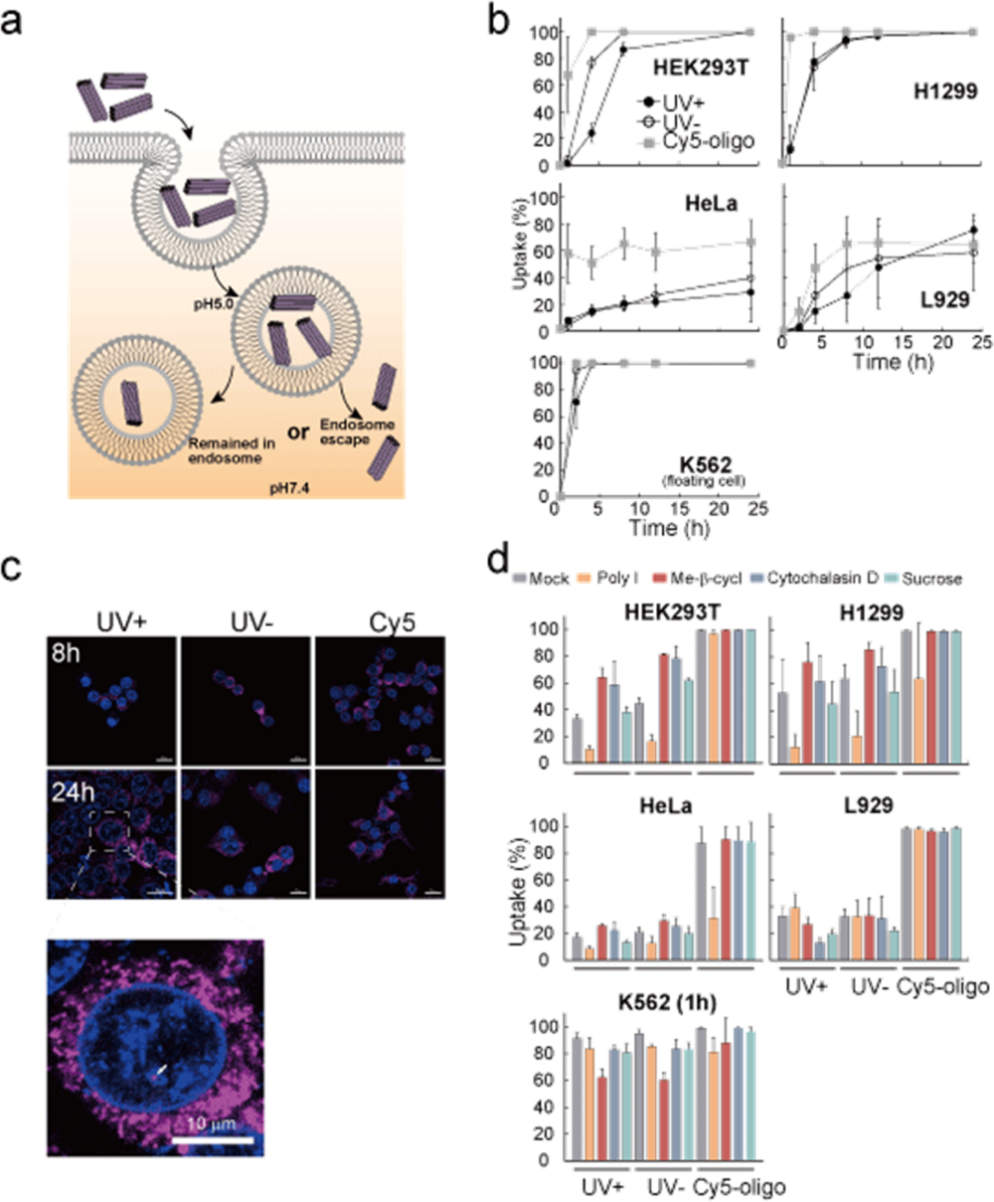
Cellular uptake of DNA origami. (a) Schematic illustration of the experiment evaluating the cell uptake of Cy5-42hb. Three samples (UV cross-linked (UV+, tough origami), without UV cross-linked (UV−), and Cy5 oligo) were infused into the cell culture medium (final concentration 10 nM). (b) Flow cytometry analysis of the cell uptake. Five cell lines were used (HEK293T, a human kidney cell line; H1299, a human lung carcinoma cell; HeLa, a human cervical cancer cell; L929, a mouse fibroblast cell; K562, a human leukemia cell). Error bars indicate the standard deviations of three to four independent experiments. (c) Representative confocal microscopy images of 42hb (UV+/−) and Cy5-oligo in HEK293T cells (8 or 24 h incubation). Although the UV+ signal was weaker than those of UV– and Cy5-oligo at 8 h, sufficient signal was observed at 24 h. Moreover, Cy5 signal in the nucleus was observed (arrow). Blue: DAPI; red: Cy5. Scale bars: 20 μm for upper images and 10 μm for zoomed image (lower image). (d) To identify the cell uptake pathway, we supplemented inhibitors (Poly I for scavenger receptors, methyl-beta-cyclodextrin for caveolin-dependent endocytosis, cytochalasin D for nonreceptor-mediated endocytosis, and sucrose for clathrin-dependent endocytosis) into the cell culture medium and examined cell uptake at 4 h (HEK293T/H1299/HeLa/L929) or 1 h (K562). Mock represents the negative control group without inhibitor treatment. Error bars indicate the standard deviation of three independent experiments.

Having made DNA origami tough through UV cross-linking and confirming the nontoxicity of tough DNA origami, we next attempted origami encapsulation into LNPs. We used lung-targeting LNPs.^13,25^ We mixed traditional LNP components (DMG-PEG, DOPE, C12-200, and cholesterol) and sorting component (DOTAP; the ratio is DMG-PEG:DOPE:C12-200:cholesterol:DOTAP = 1.3:8:17.5:23.3:50 (mol/mol), Figure 3, Supplementary Figure 6). After mixing lipids with DNA origami, we confirmed the formation of nanometer-sized particles (Figure 3a, Supplementary Figure 7). After the LNPs formed, we evaluated the effect of the LNP encapsulation process on DNA origami. When we ran LNPs with agarose gel electrophoresis, most of the DNA origami’s Cy5 signal remained in the well and did not enter the gel, suggesting that most of the DNA origami was encapsulated into LNPs (Supplementary Figure 8). To assess the DNA origami state after LNP encapsulation, we added triton, a detergent, to break down the LNPs and run the samples by using agarose gel electrophoresis. We found that tough DNA origami (UV+) remained intact until 48 h under both pH 3 and pH 5 encapsulation conditions (Figure 3b). By contrast, UV– were broken; presumably harsh encapsulation conditions (pH 3 and 5) unfolded the DNA origami structure, emphasizing the importance of UV cross-linking to tolerance for harsh conditions. Next, we confirmed the cell uptake of LNP-encapsulated DNA origami *in vitro*. The effects of encapsulation depend on the cell line (Figure 3c,d). In some cell lines, the uptake speed was increased (HeLa), some were similar (H1299), and some decreased (HEK293T). Combining with inhibitor data (Supplementary Figure 9), these data suggest that for *in vitro* cell uptake, the uptake processes were governed by cell characters, but not by LNP characters.

**Figure 3:**
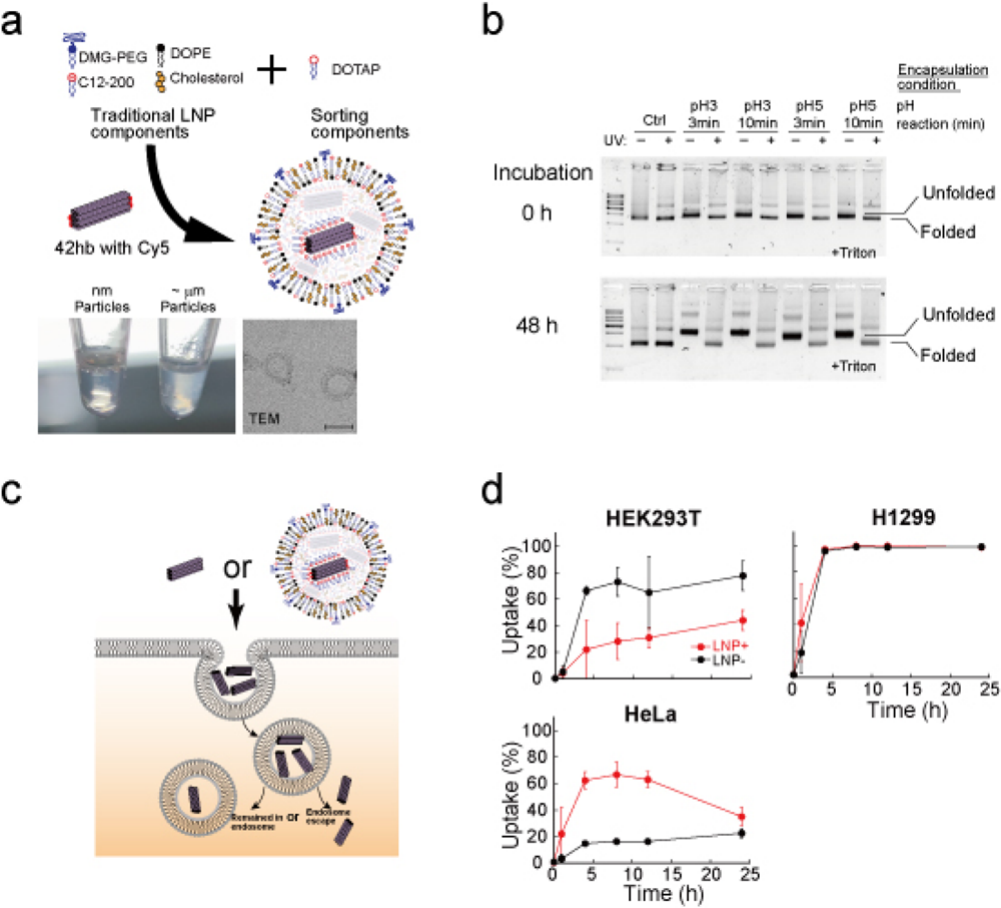
LNP encapsulation of DNA origami. (a) Schematic drawing explaining the structure of the LNP. We mixed traditional LNP components (DMG-PEG, DOPE, C12-200, and cholesterol) with sorting components (DOTAP). The solutions containing well-encapsulated nanometer-sized LNPs were clear and transparent. DLS (Supplementary Figure 7) and TEM (inset, scale bar = 200 nm) confirmed the formation of nanometer-sized particles. (b) Agarose gel electrophoresis of LNPs after treatment with triton, a detergent, to break down the LNPs. The two lines indicate the folded and unfolded 42hb positions. Ctrl, 42hb UV+/– without LNP encapsulation. (c, d) Schematic illustration (c) and data (d) of the experiment evaluating the cell uptake of Cy5-42hb UV+ encapsulated with (LNP+) or without LNP (LNP−). Samples were infused into the cell culture medium (final DNA origami concentration ∼0.21 nM, corresponding to 0.1 μg/well). Error bars indicate the standard deviation of three to five independent experiments.

The problem of *in vivo* circulation of DNA origami is easily cleared by the liver/kidney and externally discharged from the urine.^11^ As previously reported, we found that naked (non-LNP-encapsulated) DNA origami, even UV cross-linked tough DNA origami, was externally discharged within 4 h after tail-vein injection (Supplementary Figure 10). We hypothesized that if we encapsulate DNA origami by organ-targeting LNPs, the speed of accumulation in the target organ might be faster than the clearance speed (Figure 4a). As expected, DNA origami, labeled with Cy5 fluorescent dye, accumulated in the target organ (lung) within 2 h, whereas signals of naked (non-LNP-encapsulated) DNA origami were observed in only the liver and kidney with a small amount (Figure 4b). We found that even encapsulated UV– DNA origami signals were mostly lost from the target organ (lung) within 1 h, whereas UV+ tough DNA origami could be maintained in the lung for more than 4 h (Figure 4c,d), showing that UV cross-linking is important for maintaining origami in the target organ. Therefore, we focused on UV+ tough DNA origami for the following experiments.

**Figure 4:**
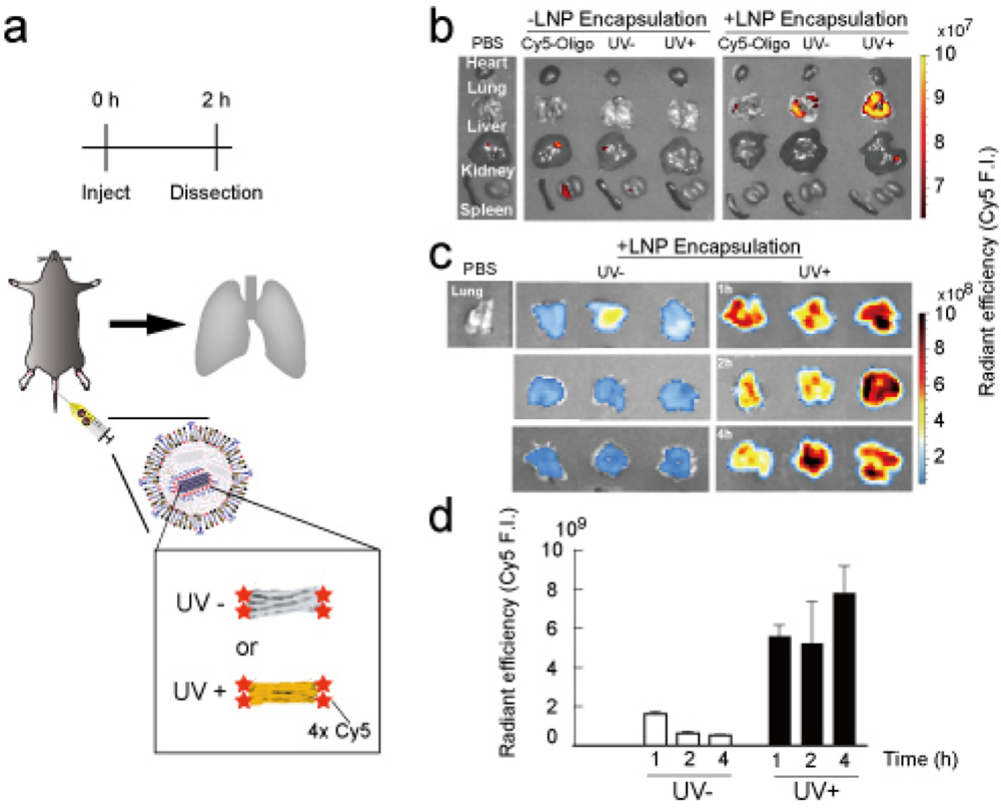
Lung delivery of LNP encapsulated in DNA origami. (a) Schematic illustration of the experiment examining the lung-specific targeting of LNP encapsulated in 42hb. DNA origami (UV+/– ) was introduced to the mice through tail vein injection at a total nucleic acid amount of 1 mg/kg. (b) Images of dissected organs (heart, lung, liver, kidney, spleen) after 2 h of tail vein injection. Only LNP-encapsulated 42hb was observed in the lungs, whereas naked-DNA origami (even UV+) was mostly cleared from the body (see also Supplementary Figure 10). (c, d) Images (c) and data (d) of dissected lung 1, 2, or 4 h after tail vein injection. UV+ DNA origami was retained more in the lungs, whereas UV– DNA origami was mostly cleared within 2 h. Error bars indicate the standard deviation of three independent mice.

We next evaluated how DNA origami affects the gene expression of LNP-encapsulated mRNA (Figure 5). We compared luciferase expression levels of three conditions (weight ratio of nucleic acids to lipid was maintained at 1:20): 1) mRNA-encapsulated LNPs (mRNA-LNPs), 2) co-delivery of mRNA-LNPs and DNA origami-encapsulated LNPs (origami-LNPs), and 3) co-encapsulation of mRNA and DNA origami into the same LNPs (mRNA/origami-LNPs). We tail-vein injected LNPs, and after 6 h, we injected luciferin, a substrate of the luciferase enzyme, and live-imaged. Then, we dissected the mice and observed the luminescence in their organs (heart, lung, liver, spleen, and kidney). To our surprise, we found that DNA origami co-delivery and co-encapsulation improved the gene expression of luciferase mRNA in the lung (Figure 5b), which was also observed with another DNA origami structure (triangular-shaped DNA origami, T1M, Supplementary Figures 11–13 and Supplementary Table 2).^26^ To confirm the reason for this enhancement effect, we performed *in vitro* cell culture experiments (Supplementary Figures 14–16). Quantitative PCR (qPCR) experiments and Luc luminescence observation showed that co-delivery and co-encapsulation of UV cross-linked tough DNA origami improved the amount of luciferase mRNA inside cells and Luc expression, presumably by enhancing endosomal escape efficiency (Supplementary Figures 14b and 15). Further research into the precise mechanism should be conducted in the future (see also Supplementary Figures 19).

**Figure 5:**
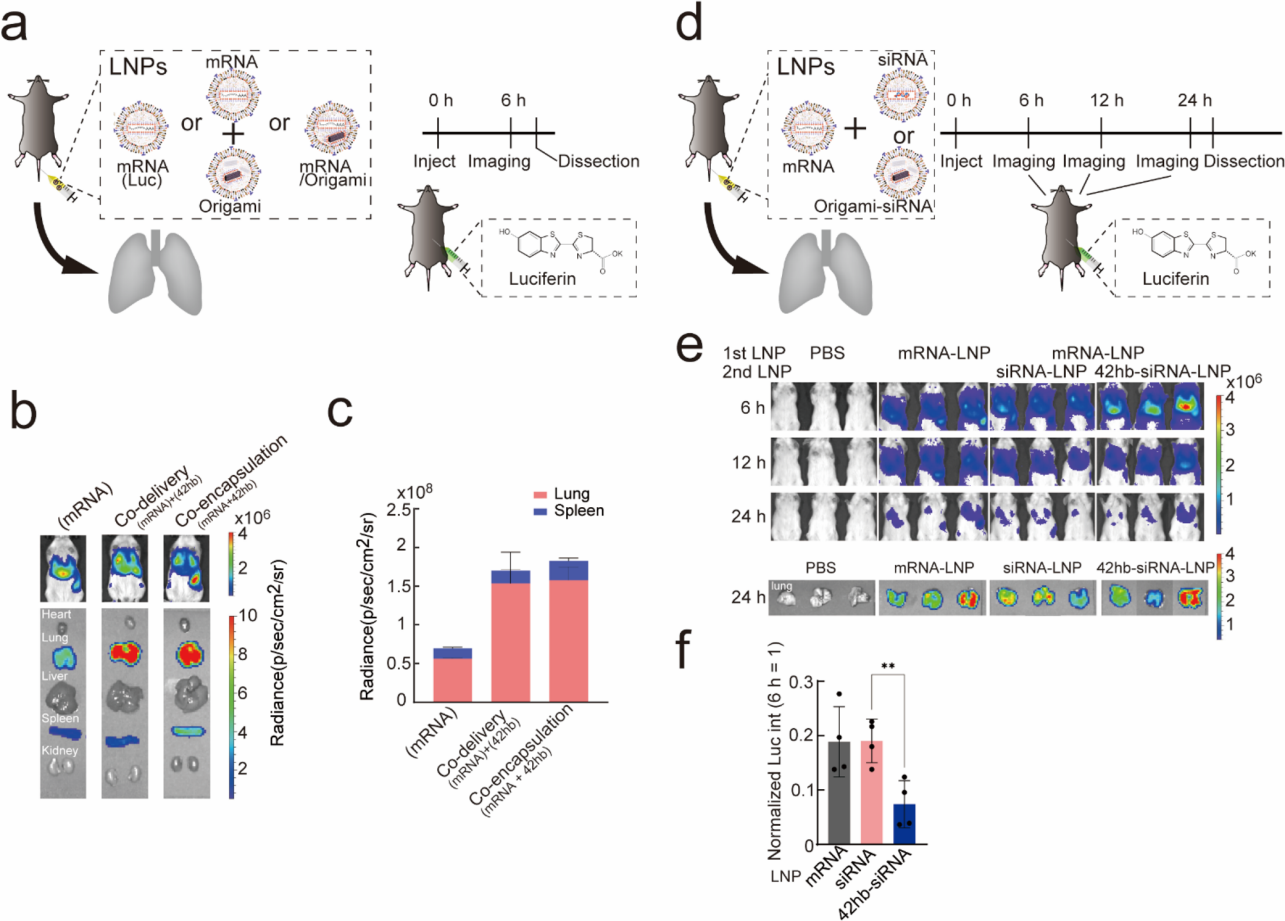
Enhanced expression and gene expression control in lung. (a) Schematic illustration of the experiment examining the effects of DNA origami on mRNA-initiated gene expression. The luciferin–luciferase (Luc) system was used. LNP encapsulated with mRNA alone, co-delivery of mRNA and 42hb, and co-encapsulation of mRNA and 42hb were compared. Luciferin was injected intraperitoneally 6 h after the first injection, and live images were obtained 6 min after luciferin injection. The mice were then dissected, and the organs were imaged 15 min after luciferin injection. (b) Live (upper) and dissected (lower) images. (c) Quantification of (b). Error bars indicate standard error of the mean (SEM) of two independent mice. (d) Schematic illustration of the experiment examining the effect of DNA origami nanodevices on knockdown (KD) efficiency in the lungs. LNP encapsulating mRNA alone was co-delivered with LNP containing siRNA alone or with siRNA integrated onto DNA origami nanodevices. The amounts of mRNA and siRNA were set to 0.2 and 0.02 mg/kg, respectively. 42hb-siRNA amount was set to 0.36 mg/kg (= 0.02 mg/kg siRNA + 0.34 mg/kg DNA origami). Luciferin was injected intraperitoneally at 6, 12, and 24 h after the first injection, and live images were captured 6 min after luciferin injection. After 24 h the mice were dissected, and the organs were imaged 15 min after luciferin injection. (e) Live (upper) and dissected (lower) images. (f) Quantification of (e). Luminescence intensities of 24 h were normalized by those of 6 h for each condition. Error bars indicate the standard deviation of four independent mice. **p < 0.01 [*t* test].

Based on the surprising effect of DNA origami on mRNA expression *in vivo*, we reasoned that DNA origami might also help the knockdown (KD) efficiency of siRNA (Figure 5d–f). We attached siRNAs against luciferase mRNA through 32 hybridization sites (origami-siRNA, Supplementary Figures 17 and 18 andSupplementary Tables 3 and 4) and encapsulated them into lung-delivery LNPs. We compared the origami-siRNA LNPs with siRNA-only encapsulated LNPs. We found that the KD efficiency of origami-siRNA was higher than that of siRNA-only, whereas the KD efficiency of siRNA-only is similar to that of siRNA-without delivery (NC condition in Figure 5f, delivery-only mRNA), indicating that DNA origami indeed helps to improve the KD efficiency of siRNA. Combined with the enhancement effect of DNA origami on mRNA expression, DNA origami might have some unknown positive effect on the function of the delivered gene of interest (i.e., mRNA and siRNA). To uncover the molecular mechanism behind this positive effect, we performed cryogenic electron microscopy. We found that DNA origami related LNP structures were different from those of mRNA-alone and siRNA-alone LNPs (Supplementary Figures 19 and 20). Considering the literature report that LNP structure correlates with the efficiency of endosomal escape and subsequent GOI function, we hypothesized that the different LNP structures of DNA origami-related LNP contribute to the enhancement of GOI function.^27^ The unique surface charge situation of DNA origami and other factors might also contribute to this gene function enhancement.^17−19^

To demonstrate the versatile ability of the LNP-encapsulated origami approach, we switched the lung-targeted LNPs to liver-targeting LNPs. We used an optimized composition of conventional LNPs.^13^ As expected, origami improved mRNA expression, and origami-siRNA LNPs also enhanced the KD efficiency of siRNA (Supplementary Figure 21); we noted that some signals leaked into the spleen owing to the imperfect targeting capability of liver-targeting LNP). Lung and liver data showed that origami can enhance the gene expression activity of delivered GOI by simply changing the components of LNPs.

Here, we used lipid nanoparticle technology to overcome the limitation of DNA origami, which is easily cleared by the liver/kidney and externally discharged from urine. As proved by the COVID-19 mRNA vaccine, the current advancement of LNP technology, such as specific organ targeting, is phenomenal, and it is a straightforward approach to apply this LNP technology to DNA origami. However, the harsh encapsulation conditions of LNP encapsulation have hindered its application. Our results showed that UV cross-linking of DNA origami helped overcome these challenges and also provides unexpected positive effects on the activity of delivered GOI. Although Cryo-EM data showed the contribution of different LNP structures, the detailed molecular mechanism should be addressed in future studies to harness these positive effects to realize nanodevices functioning inside individual animals. The unique surface charge,^17−19^ negative charge,^28^ and/or the large size of the DNA origami^27^ might contribute to this positive effect, which might assist endosomal escape of the GOI. The recent impressive effect of protein of interest (e.g., Cas9) delivery toward tumors might also use a similar mechanism, predicting the bright future of DNA origami-based nanodevices.^8−10^ Moreover, combining DNA origami capability proved *in vitro*, i.e., computational capability,^29^ target-specific binder capability,^30^ and autonomous gene expression capability,^22^ will allow us to manufacture nanodevices that can compute and control temporal-spatial gene expression.

## Supporting Information Available

DNA origami preparation; UV irradiation; gel electrophoresis of DNA origami; cryo-electron microscope; DNA origami stability verification *in vitro*; cell culture, cell uptake (including inhibitor experiments), and cell viability check; DNA origami-LNP preparation; UV-irradiated origami circulation in mice; LNP-origami circulation in mice; enhanced mRNA expression in the lungs by origami-LNP; siRNA release by RNase H; 42hb-siRNA origami preparation; qPCR to quantify the mRNA amount after cell uptake; evaluating lysosomal escape using a confocal microscope; Luc knockdown experiment using 42hb-siRNA origami *in vivo* (mice); Luc knockdown experiment using 42hb-siRNA origami *in vitro* (culture cells); Cryo-EM structures of LNPs; gene expression control in liver (PDF)
