# Supplementary material for: Organ-Specific Gene Expression Control Using DNA Origami-Based Nanodevices
Source: Nano Lett. 2024 Jun 26;24(27):8410–7. doi: 10.1021/acs.nanolett.4c02104 (PMC11249008; doi:10.1021/acs.nanolett.4c02104)
Supplement: Supplementary file 1 — nl4c02104_si_001.pdf [file nl4c02104_si_001.pdf]

Supplementary Information for

Organ-specific gene expression control using DNA origami-based nanodevices

Yuxiang Liu [1,#], Ruixuan Wang [1,#], Qimingxing Chen [1], Yan Chang [1], Qi Chen [1], Kodai Fukumoto [2,3], Bingxun Wang [1], Jianchen Yu [1], Changfeng Luo [1], Jiayuan Ma [1], Xiaoxia Chen [1,4], Yuko Murayama [3], Kenichi Umeda [5], Noriyuki Kodera [5], Yoshie Harada [2], Shun-ichi Sekine [3], Jianfeng Li [1,6*] and Hisashi Tadakuma [1,6*]

[1] School of Life Science and Technology, ShanghaiTech University, Shanghai 201210 People's Republic of China
[2] Institute for Protein Research, Osaka University, Osaka 565-0871, Japan
[3] RIKEN Center for Biosystems Dynamics Research, Yokohama 230-0045, Japan
[4] Zhejiang University School of Medicine First Affiliated Hospital, Zhejiang Provincial Key Laboratory of Pancreatic Disease Hangzhou, Zhejiang, 310009, China
[5] Nano Life Science Institute (WPI-NanoLSI), Kanazawa University, Kakuma-machi, Kanazawa, 920-1192, Japan
[6] Gene Editing Center, School of Life Science and Technology, ShanghaiTech University, Shanghai, 201210, China
[#] Y.L and R.W contributed equally to this paper
[*] Corresponding Authors: tadakumahisashi@shanghaitech.edu.cn, lijf1@shanghaitech.edu.cn

This file includes:

Supplementary Material and Methods
Supplementary Figures (Supplementary Fig. 1 to 21)
Supplementary Tables (Supplementary Table 1 to 4)

Supplementary Material and Methods

1. DNA origami preparation

A DNA origami rod (42 helix-bundle, 42hb) was designed based on a previous report[20] using caDNAno (ver 2.3.0). Single-stranded p7560 was purchased from Tilibit nanosystems and used as a scaffold for DNA origami. Unmodified staple strands were purchased from GenScript as Reverse Phase Cartridge (RPC) grade. Fluorescent dye (Cy5) modified staples were purchased from GenScript as HPLC grade (see **Supplementary Table. 1–3** for the detailed staple information).

The DNA origami rod was folded in 1× folding buffer (5 mM Tris boric acid, pH 7.6, 1 mM EDTA, 5 mM Na(OAc) and 20 mM $Mg(OAc)_2$). Generally, 20 nM single-stranded p7560 (Tilibit) and 200 nM of each staple strands (10 fold) were mixed in 1× folding buffer and folded under the program at 65°C for 15 min and then 50°C for 8 h.

Then, the DNA origami was purified using PEG precipitation, and 15% (W/V) PEG (with 5 mM Tris-borate, 1 mM EDTA, and 500 mM NaCl) was mixed with the sample at a ratio of 1:1. The mixture was placed at room temperature for 10 min and then centrifuged at 16,000 g, 25°C for 30 min. The supernatant containing excess staple strands was removed, and the precipitate was dissolved in 1× folding buffer (containing 20 mM $Mg(OAc)_2$).

2. UV irradiation

We used a compact xenon light source (MAX-350, Asahi Spectra) with a high transmission bandpass filter centered at approximately 310 nm (LX0310, Asahi Spectra, half maximum full-width of 10 nm). To ensure the stability and homogeneity of

irradiation, we used a power stabilizer unit (FBU-10, Asahi Spectra) and rod-lens (RLQL80-1, Asahi Spectra), respectively. Briefly, samples were placed right below the light guide (rod-lens), and the distance was adjusted to around 50 mm (irradiation area is ~□10 mm = 1 cm^2). Unless otherwise indicated, the light intensity was set and stabilized at 1500 μW using optical power meter (AQ2150A, Ando Electric). The samples were irradiated for 60min at room temperature in 1× folding buffer.

To label DNA origami with Cy5 (Cy5-Origami), two or four staples with 5'-Cy5 were purchased from GenScript. To incorporate the Cy5-staples, firstly, DNA origami was folded using a staple mix omitting Cy5-staples and purified by PEG precipitation. Then, a 5-fold excess of Cy5 staples was annealed to the UV-irradiated origami (for UV+ samples) or non-UV-irradiated origami (for UV- sample) at 45°C for 1 h followed by one-time PEG precipitation to remove excess Cy5-staples.

To label siRNA integrated DNA origami (42hb-siRNA), Cy5 was introduced by the guide RNA sequence of the siRNAs (see below section "12. siRNA release by RNase H" for details).

3. Gel electrophoresis of DNA origami

Samples were electrophoresed on 1% agarose gels (Bio-rad) containing 0.5 × Tris-borate-EDTA and 5 mM $MgCl_2$ for 40 min at 100 V and 4°C. The running buffer was 0.5 × tris-borate-EDTA with 2 mM $MgCl_2$. After staining with SYBR safe (Invitrogen), the electrophoresed agarose gels were imaged using Amersham ImageQuant 800 (Cytiva) and the resulting images were analyzed using ImageJ.

4. Cryo electron microscopy (Cryo-EM) for 42hb origami

Approximately 1 μM of DNA origami was applied to the Quantifoil R1.2/1.3 copper grid with 300 meshes. The grids were plunge-frozen in liquid ethane using an EM GP2 (Leica), with a blotting time of 2–3 s at 75% humidity and 10°C. Cryo-EM images were collected using a 200 kV Tecnai Arctica transmission electron microscope (FEI) equipped with a K2 summit direct electron detector (Gatan) in electron counting mode. The movies were recorded at a total exposure dose of 50 e-/Å2 with a raw pixel size of 1.47 Å.

The 522 recorded movies were imported and all subsequent processing steps were performed using cryoSPARC (v3.2.0). After patch motion correction and patch CTF estimation, a few thousand particles were manually picked to create templates for template picker. The autopicked particles were then extracted from the micrographs and subjected to multiple rounds of 2D classification to remove falsely picked grid contaminations and damaged particles. The best 2D class averages, determined by visual inspection, were selected and used in the Ab-3D-initio reconstruction. After two times 3D reconstructions, the result was used to perform a one-time 3D non-uniform refinement and exported to Chimera X for visualisation (v1.2.5).

5. DNA origami stability verification *in vitro*

To examine the stability of DNA origami with or without UV crosslink (UV- and UV+), 1 μM DNA origami (UV- or UV+) were dissolved in 1× folding buffer containing different Mg concentrations (0 to 20 mM). DNA origami samples were incubated at room temperature (25°C) for 1 month to check their DNA origami status by gel electrophoresis. For thermal stability check, 10 nM DNA origami (UV- or UV+) were incubated in 1× folding buffer at different temperature (40 to 90°C) for 30 min in a thermal cycler machine

(ProFlex PCR System, Thermo). For stability checks under cell culture conditions, 10 nM DNA origami (UV- or UV+) was buffer exchanged from 1× folding buffer to PBS + 10% FBS by PEG precipitation. Samples were incubated in a thermal cycler machine at 37°C until the designated time point. Then, the reactions were quenched by the addition of 4 × loading buffer (40% glycerol, 4 mM EDTA, and 80 mM $Mg(OAc)_2$) and applied to agarose gel electrophoresis. For DNase stability check, 10 nM DNA origami (UV- or UV+) were buffer exchanged from 1 × folding buffer to 1 × DNase I buffer (10 mM Tris-HCl, pH 7.6, 2.5 mM $MgCl_2$, and 0.5 mM $CaCl_2$) by PEG precipitation. DNase I (NEB) and BSA (Takara) with a final concentration of 0.2 U/mL and 0.01% were added, and the mixture was incubated in a thermal cycler machine at 37°C. Once the time point was reached, 4 × loading buffer (40% glycerol, 20 mM Tris boric acid, pH 7.6, 4 mM EDTA, 20 mM Na(OAc), 80 mM $Mg(OAc)_2$, 0.1% BPB and 0.36% SDS) was immediately added and the samples were transferred to 4°C refrigerator to stop the reaction. For the pH stability check, 10 nM DNA origami (UV- or UV+) was buffer exchanged from 1× folding buffer to PBS (pH 7.4 or 5.0, without supplemental Mg^{2+} ion) by PEG precipitation. Samples were incubated in a thermal cycler machine at 37°C for 0, 1, 2, and 4 h. Then, the reactions were quenched by the addition of 4 × loading buffer (40% glycerol, 4 mM EDTA, and 80 mM $Mg(OAc)_2$) and applied to agarose gel electrophoresis.

ImageJ was used to quantify the gel band intensity, and Excel, GraphPad Prism, and/or KaleidaGraph were used to analyze and draw graphs.

6. Cell culture, cell uptake (including inhibitor experiments), and cell viability check

Cell lines (HEK293T, a human kidney cell line; H1299, a human lung carcinoma

cell; HeLa, a human cancer cell; L929, a mouse fibroblast cell; K562, a human leukemia cell) were cultured in Dulbecco's Modified Eagle Medium (DMEM) (Life/Invitrogen) with 10% fetal bovine serum (FBS) (v/v) (Gibco or Lonsera). The cells were cultured at 37℃ with 5% CO_2.

For cell uptake analysis using FACS, cells were seeded on poly-L-lysine (Sigma) pre-coated (for adherent cells: HEK293T, H1299, HeLa, and L929) and non-coated (for floating cells: K562) 96-well plates (Titan). After 12 h incubation at 37℃ with 5% CO2, 5 μL of 200 nM Cy5-Origami was added into 95 μL of culture medium (final Cy5-Origami concentration is 10 nM). After incubating the cells with DNA origami for designated incubation times (0, 1, 4, 8, and 24 h or 0, 2, 4, 8, 12, and 24 h), the cells were washed twice with PBS, harvested, and subjected to flow cytometry (FACS, BD, LSRFortessa, APC channel). Flow Jo software was used to calculate the percentage of Cy5 positive cells and quantify the signal intensity.

To analyze the cell uptake mechanism, we used four different inhibitors of specific cell endocytic pathways. Polyinosine (Poly-I, 40 μg/mL, 30 min pretreatment) (Sigma) can bind and saturate scavenger receptors; Cytochlasin D (CytoD, 0.25 μM, 15 min pretreatment) (Abcam) inhibits the non-receptor mediated endocytosis; Methyl-cyclodextrin (Me-cycl, 625 nM, 30 min pretreatment) (Sigma) inhibits the caveolin-dependent endocytosis; Sucrose (100 mM, 30 min pretreatment) (Beyotime) inhibits the clathrin-dependent endocytosis. After pretreatment with the inhibitors, the cells were incubated with Cy5-DNA origami (10 nM) for 4 h and analyzed using FACS.

For confocal experiments, HEK293T cells were incubated with Cy5-Origami (final 10 nM) in an 8-well glass chamber (cellvis) for 8 and 24 h. Cells were washed twice with PBS and stained with Hoechst 33324 (Sigma) for 20 min, then washed three times with

PBS, and 200 µL of DMEM with 10% FBS was added before imaging with a confocal microscope (Zeiss 800).

For cell viability, Cell Counting Kit-8 (CCK-8, TargetMol) was used. HeLa cells were washed twice with PBS. Then, 100 µL of CCK-8 assay solution (90 µL PBS + 10 µL CCK-8 solution) was added into the well. After another 40 min of cell culture, the absorbance at 450 nm was measured using plate reader (Tecan-Spark). The OD of the naïve control group that received no treatment was used as the standard for normalization.

7. C12-200 synthesis

C12-200 was synthesized based on the previous literature (ref#25, Melamed et al., *J Control Release*. **2022**, 341, 206-214. doi: 10.1016/j.jconrel.2021.11.022). The amine N1-(2-(4-(2-aminoethyl)piperazin-1-yl)ethyl)ethane-1,2-diamine (50mg, 1eq) was reacted with the tail 2-decyloxirane (354ul, 7eq) at 90℃ for 3 days to form the lipidoid C12-200. The residue was purified by silica gel column chromatography (Methanol/DCM 1:1 to 3:1) to give C12-200 (200 mg, 76%). The obtained product of C12-200 was determined by ^{1}H NMR in CDCl3: ^{1}H NMR (500 MHz, CDCl3) δ 3.70 – 3.52 (m, 5H), 3.51 – 1.51 (m, 31H), 1.43 (m, 10H), 1.26 (m, 80H), 0.88 (t, J = 6.9 Hz, 15H). m/z: [M+H] + calculated for C70H145N5O5 1137.12; found 1137.093.

8. DNA origami-LNP preparation

DNA origami was prepared and encapsulated in lipids to form a lipid nano particle (LNP). The lung-targeting LNP contained DMG-PEG (MACKLIN), C12-200 (CAS#1220890-25-4, synthesized), DOPE (AVT), cholesterol (Admas) and DOTAP (Avanti), whereas the liver-targeting LNP contained the remaining four components

except for DOTAP. The origami to lipid ratio during encapsulation was 1:20. For encapsulation, 25 mM sodium acetate (pH5) was prepared for dissolving DNA origami in one tube, while the lipids were mixed in ethanol in another tube. Solutions in two tubes were mixed and pipetted thoroughly to achieve the encapsulation reaction. Following the encapsulation reaction, the LNP-containing buffer was exchanged to PBS using dialysis. The characteristic of the LNP were measured using a zetasizer (Nano-ZS, Malvern) and agarose gel electrophoresis.

9. UV irradiated origami circulation in mice (related to Supplementary Fig. 10)

To reduce the autofluorescence of Cy5-channel, ICR mice (aged 3-4weeks, Lingchang) were fed chlorophyll -free diets (D10001, Research Diets) for at least one week. UV+ DNA origami labeled with 4 × Cy5 was intravenously injected into the mice using the visual injection platform (YAN-Q9, from Shanghai Yuyan Instruments) at a dose of 500 nM in 200 μL. Live images were taken at designated time points to trace the distribution of Cy5-Origami. The IVIS Spectrum Series (PerkinElmer) was used to perform live imaging and 2% isoflurane (RWD Life Science) was delivered using the nose cone before and during the imaging process to keep the mice anesthetized.

10. LNP-origami circulation in mice

To reduce the autofluorescence of Cy5-channel, ICR mice (aged 3-4weeks, Lingchang) were fed chlorophyll -free diets (D10001, Research Diets) for at least one week. UV- or UV+ LNP was injected into these mice intravenously (i.v.) at a dose of 1 mg/kg. The IVIS Spectrum Series (PerkinElmer) was used to perform live imaging and 2% isoflurane (RWD Life Science) was delivered through the nose cone before and during

the imaging process to keep the mice anesthetized. The mice were dissected at predetermined time points and the heart, lungs, liver, spleen and kidneys were imaged.

11. **Enhance mRNA expression in the lung by origami-LNP (related to Fig. 5)**

ICR mice (aged 4 weeks) were purchased from Lingchang, Shanghai. Luciferase mRNA was transcribed from plasmid DNA through transcription, capping and poly-A tail addition (kit from Novoprotein). The mRNA and UV+ Origami were either co-delivered or co-encapsulated by lung-targeting LNP to the mice through tail vein injection. The mRNA dose was set to be 0.2 mg/kg and the dose of DNA origami was 0.36 mg/kg. At 6 h post injection, luciferin (15 mg/mL in PBS, MeilunBio) was injected intraperitoneally, and images of live imaging and dissected organs were obtained 6 min and 15 min after luciferin injection using the IVIS Spectrum Series (Perkinelmer).

12. **siRNA release by RNase H (related to Supplementary Fig. 18)**

Staples corresponding to 32 sites on the DNA origami (see **Supplementary Fig. 1 and Table 3)** were elongated with an extra DNA capture strand with the sequence : GTGCTACTCCAGTTCTTT. The siRNA passenger strand was elongated using an additional RNA anchor strand with the sequence: GAACUGGAGUAGCACAAA. These captured passenger strands can form DNA-RNA hybrid that can be specifically digested by RNase H. The 3′ of the guide strand was modified with Cy5 molecule to serve as an indicator. The staple capture strand, passenger strand and guide strand were mixed and annealed together in 1× CutSmart buffer (NEB), then cleaved by RNase H (ABclonal) under different doses (2.5, 5, 7.5, or 10 U) at 37°C for 1 h.

13. **42hb-siRNA Origami (siRNA integrated DNA origami) preparation**

UV+ 42hb was first prepared at a concentration of 20 nM in 1× folding buffer. The siRNA was ordered as two strands of ssRNA from GenScript with the following sequences: Passenger-GAACUGGAGUAGCACAAAGAUUGACAAAUACGAUUUAUC and Guide-UAAAUCGUAUUUGUCAAUCAG. The siRNA was annealed in CutSmart buffer (NEB) and mixed with 42hb at a molar ratio of 10:1. The mixture was incubated at 4°C overnight. Excess siRNA was removed by PEG precipitation and the 42hb-siRNA was dissolved in 1× folding buffer at the required concentration.

14. **Macropinocytosis pathway analysis (dextran uptake, related to Supplementary Fig. 14)**

Luciferase (Luc) mRNA was labeled with Cy5-UTP as described above. Then, LNP encapsulated with 1) mRNA alone, 2) co-delivered mRNA and 42hb, and 3) co-encapsulated mRNA and 42hb, were prepared. HeLa cells (1.5×10^4 cells/well) were seeded into a 96-well glass bottom plate (Cellvis, P96-1.5H-N), and after 16 h, LNPs and FITC-dextran (Beyotime, ST2947) were added with the doses of 0.1 µg, 0.18 µg, and 0.2 mg/mL for mRNA, 42hb, and FITC-dextran, respectively. Thirty minutes after incubation, culture medium was removed and cells were washed three times with pre-cooled PBS. Then, 100 µL/well 4% PFA was added, and cells were fixed at R.T. for 15 min. PFA was removed followed by 3 washes with pre-cooled PBS. FluoroBrite DMEM (Thermo) containing 10% FBS was added to the cells for confocal imaging (Nikon TI2-E +CSU W1 Sora 1Camera). The green channel (561 nm) represents FITC-dextran,

indicating the location of macropinocytosis, whereas the red channel (640 nm) represents Cy5-mRNA. The co-localization of Mander's coefficient was calculated as described below.

15. **Quantification of co-localization using the Mander's coefficient method (related to Supplementary Figs. 14&15)**

Acquired images were applied to the same look-up table (LUT), and each color channel image was saved as an 8-bit Tiff file using NIS-Elements Viewer (Nikon, v5.21). The images for different channels were then imported into ImageJ (v1.53c) and were merged into one image using the 'Merge Channels' option. The Mander's coefficient under the automatic threshold was then calculated using the JACoP plugin (Bolte, S. & Cordelieres, FP. *J Microsc*. **2006**, 224, 213-232). Box plots of M1 and M2 were drawn using GraphPad Prism software.

16. **Evaluating lysosomal escape using a confocal microscope (related to Supplementary Fig. 15)**

LNP encapsulated with mRNA alone, co-delivered of mRNA and 42hb, and co-encapsulated mRNA and 42hb were compared during cell uptake. To label the mRNA with Cy5, transcription reactions were performed using Cy5-UTP (APExBIO). HeLa cells (3.8×10^4 cells/well) were seeded in an 8-well chambered cover glass (Cellvis), and after 16 h, LNPs were added. mRNA and 42hb were added at doses of 0.253 μg and 0.4554 μg respectively. After 3 h of cell uptake, the cells were washed twice with PBS and stained with Hoechst 33342 (Sigma) and LysoTracker-Green (MeilunBio) for 30 min. The staining buffer was removed, and the cells were washed twice with PBS.

FluoroBrite DMEM (Gibco) containing 10% FBS was added to cells for confocal imaging (Nikon CSU-W1 with a SoRa camera). The blue channel (488 nm) represents the cell nucleus, green channel (561 nm) represents lysosomes and red channel (640 nm) represents mRNA. The Mander's coefficient was calculated and compared as described above.

17. **qPCR to quantify the mRNA amount after cell uptake (related to Supplementary Fig. 15)**

Primers for qPCR were designed using Primer Premier 6 and synthesized by GenScript as Reverse Phase Cartridge (RPC) grade. The following primers were used for GAPDH: Forward: AGAAGGCTGGGGCTCATTTG, reverse: AGGGGCCATCCACAGTCTTC, and for luciferase: Forward: CGACCAACGCCTTGATTGACAA, reverse: GGGAGCCACCTGATAGCCTTTG. After a certain time of incubation (1, 2, 4, or 6 h), HeLa cells were washed twice with PBS and lysed using TRIZOL (Thermo). RNA was extracted from the cell lysate using chloroform and purified by ethanol precipitation. cDNA was reverse-transcribed from the extracted RNA using RT Master Mix (Takara). qPCR was performed using SYBR green master mix (ABI) and QuantStudio Q3 (Thermo).

18. **Identification of elements essential for positive effect on GOI function (related to Supplementary Fig. 16)**

HeLa cells (1.5×10^4 cells/well) were seeded into a white 96-well plate with a transparent bottom (In vitro scientific) and incubated for 16h. Luc mRNA was either co-delivered or co-encapsulated with cre-mRNA, 42hb UV-, 42hb UV+, or empty LNPs.

The Luc mRNA dose was 0.1 μg and the nucleic acid (42hb and cre-mRNA) dose was 0.18 μg per well. 8 h after adding these LNPs, the culture medium was replaced with fresh medium, and the cells were further cultured for 16 h. At 24 h after LNP treatment, the culture medium was removed, and the cells were washed three times with PBS. Then, 0.15 mg/mL luciferin in PBS was added and cells were further cultured at 37℃ for 10 min, then bioluminescence was measured by plate reader (Tecan Spark).

19. **Luc knock down experiment using 42hb-siRNA Origami *in vivo* (mice)**

Luciferin-Luciferase (Luc) system was used as a reporter system. siRNA and 42hb-siRNA were encapsulated into LNP and co-delivered with Luc-mRNA-LNPs via intravenous injection. The mRNA dose was set to 0.2 mg/kg and the dose of 42hb-siRNA was 0.36 mg/kg. Luciferin was injected intraperitoneally at 6, 12, and 24 h after the first injection, and live images were captured 6 min after luciferin injection. After 24 h, the mice were dissected and the organs were imaged 15 min after luciferin injection.

20. **Luc knock down experiment using 42hb-siRNA Origami *in vitro* (culture cells, related to Supplementary Fig. 18)**

The Luc system in HeLa cells was used as the reporter system. siRNA and 42hb-siRNA Origami were encapsulated into LNP. Culture cells (1.5×10^4 cells/well) were seeded in a 96-well plate 16 h before transfection. A total of 0.1 μg mRNA and 0.06, 0.12, or 0.24 pmol siRNA (or 42hb-siRNA Origami, with the same amount of siRNA to free siRNA) were mixed and diluted to 10 μL in PBS to achieve the same final volume before being applied to the cells. To avoid the potential toxicity of LNP to cells, a fresh culture medium was replaced 8 h after transfection. After another 16 h,

luciferin (15 mg/mL in PBS) was diluted 100 times with PBS and applied to the cells. Ten minutes after incubation, luminance was measured using plate reader (Tecan-Spark).

21. **Cryo electron microscopy for LNP and data analysis (related to Supplementary Fig. 19&20)**

To prepare LNP samples for cryo-EM imaging, either Quantifoil R1.2/1.3 Cu 300 with 2 nm ultrathin carbon-coated or lacey carbon-coated 300 mesh copper grids (Ted Pella) were glow discharged with H_2/O_2 for 30 s. Then, 4 μL of sample was applied to the grids and incubated for 10 min. The grids were blotted with filter paper, and another 4 μL of sample was applied. The grids were then blotted for 2.5 s and plunge frozen in liquid ethane using Vitrobot Mark IV (Thermo Fisher Scientific) under 22℃ and 100% humidity. The grids were kept in liquid nitrogen until imaging. The images were collected on a Talos Arctica electron microscope (FEI) operating at 200 kV accelerating voltage, at a calibrated magnification of 120,000×, using a falcon III electron camera. Images were taken at -2 ~ -3 μm defocus to improve contrast and the exposure time was 1 ~ 5 s with a dose rate of 20 e^-/pixel/s.

For analysis, images were exported as tiff format and then imported into ImageJ for FFT analysis. The scale of all images was set to be 500 nm for 4096 pixels (8.192 pixels/nm). A square ROI was drawn inside a circle, and built-in function of ImageJ was used to obtain FFT images. Then, the same contrast setting was applied to all FFT results. To calculate the lattice spacing, lattice spacing of four sites of each FFT images were measured and averaged. Then, the mean and standard deviation of each structure pattern were calculated.

a

b

c

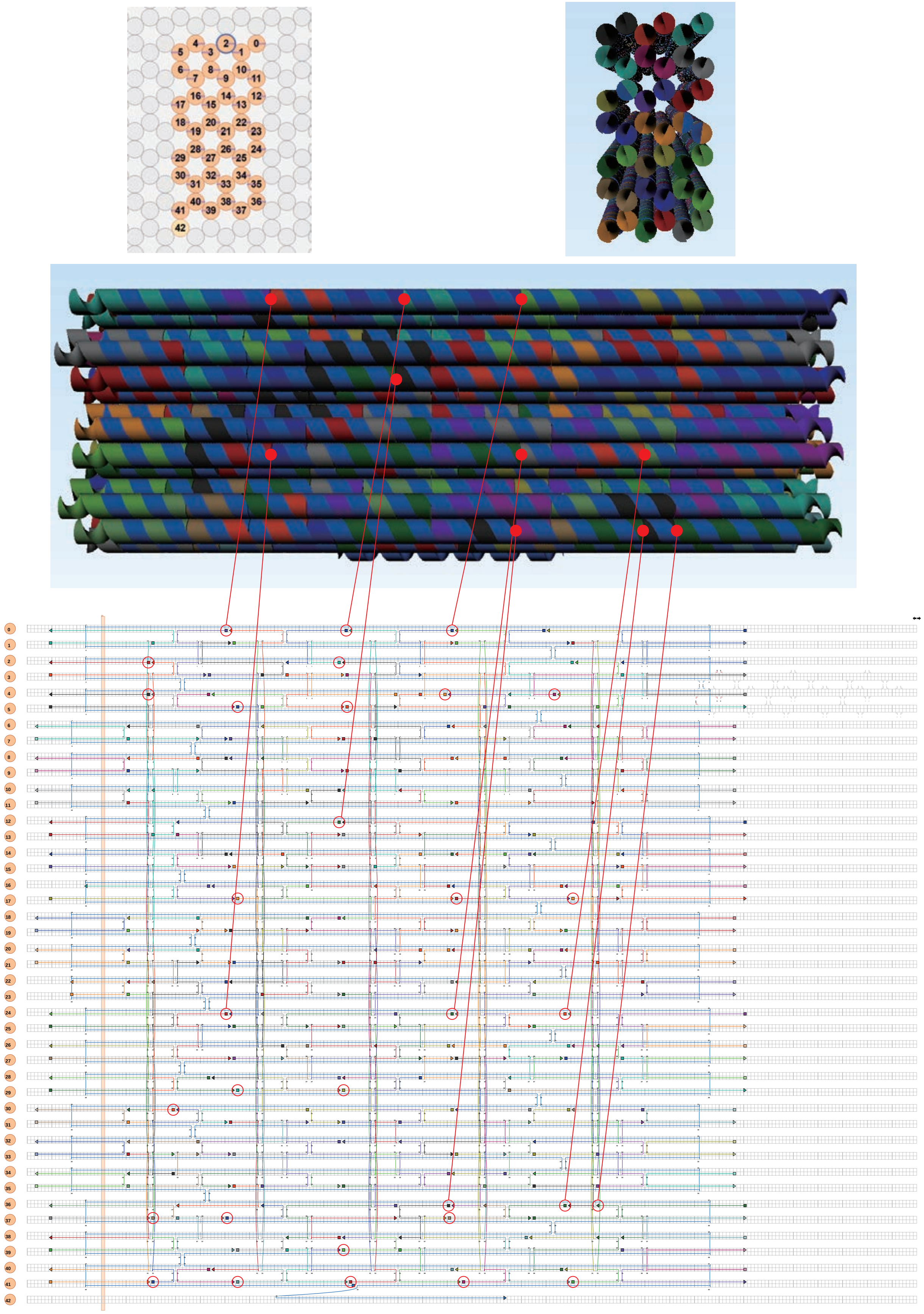

Supplementary Fig. 1. Structure and layout of the DNA rod (42hb).
(a) Cross section images of caDNAno (left) and Maya (right). (b) Side view showing the positions of the handles for siRNA anchoring (red circles). (C) Secondary structure of the rod rendered using caDNAno. Red circles indicate 32 siRNA binding sites, 10 of 32 sites are connected to the corresponding locations in (b).

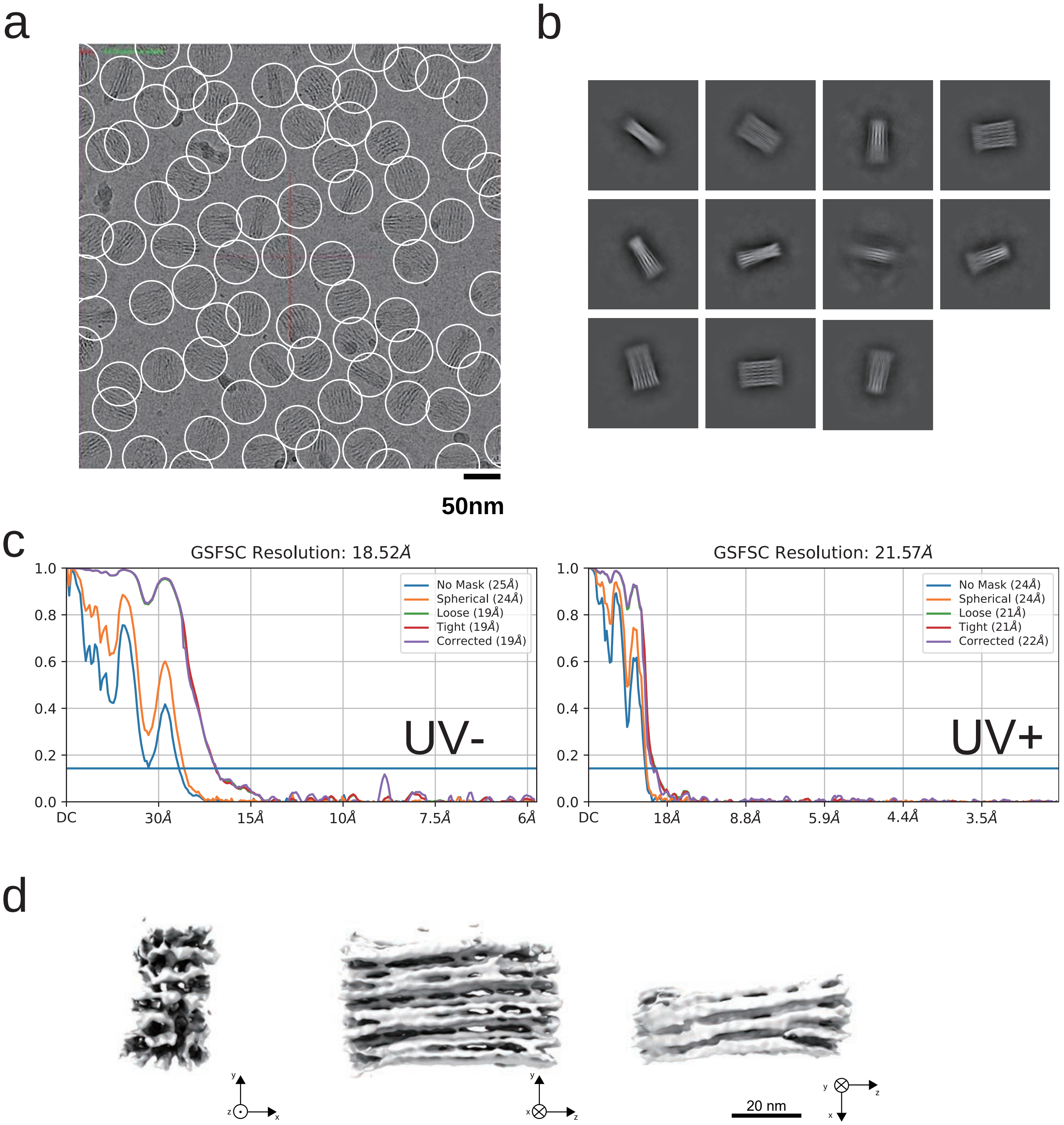

Supplementary Fig. 2. Cryo-EM analysis of DNA origami structure.
(a) Representative Cryo-EM image. Circles indicate the positions of the DNA origami rod (42hb). (b) Representative 2D class averages of 42hb UV−. (c) FSC curves showing a global resolution of 18.5 and 21.6 Å for UV− and UV+, respectively. The results were obtained from 5764 and 4632 selected particles extracted from 398 and 1170 images for UV− and UV+, respectively. (d) The r econstructed 3D density map of the 42hb UV−.

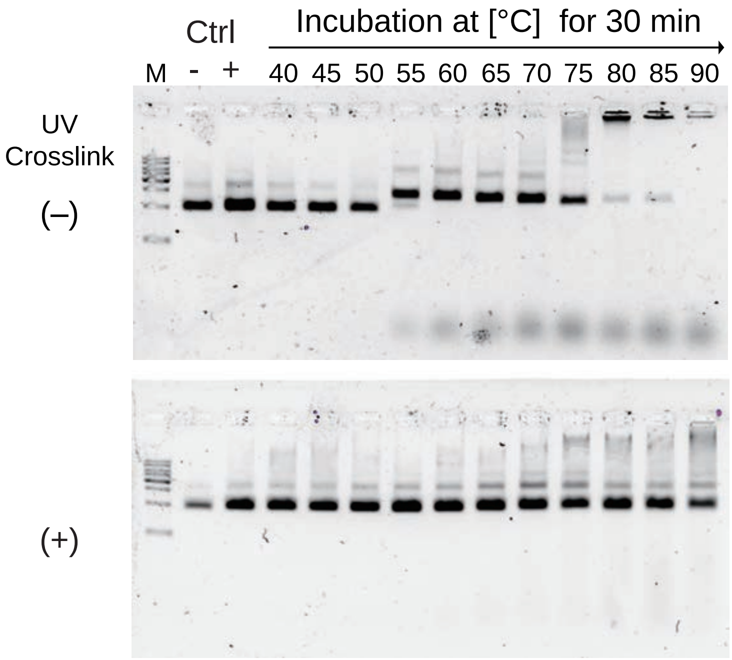

Supplementary Fig. 3. Thermal stability of DNA origami.
Agarose gel electrophoresis of 42hb incubated at high temperature for 30 min. Ctrl, 42hb UV± sample incubated at room temperature.

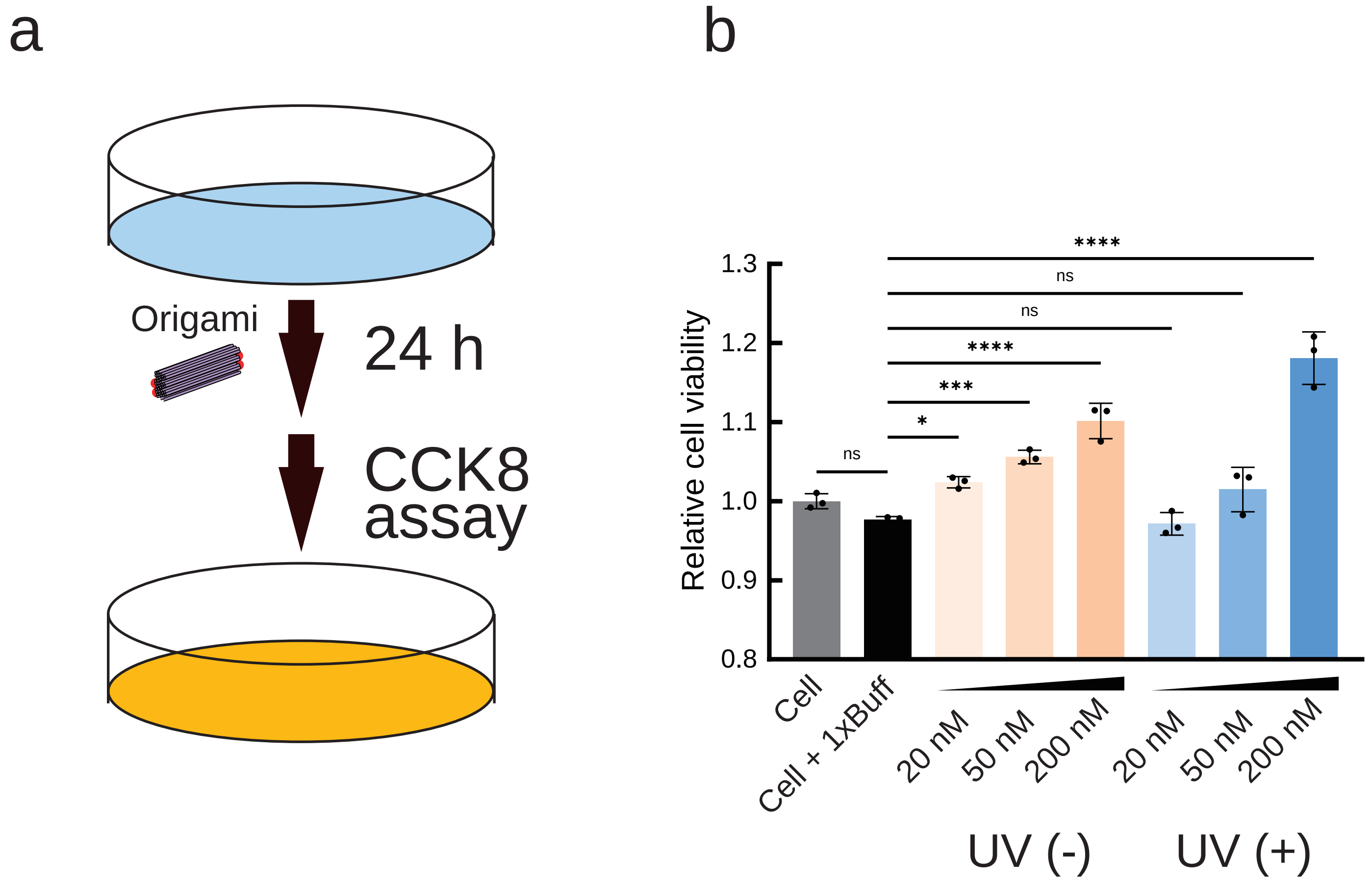

Supplementary Fig. 4. CCK8 assay to evaluate origami effect on cell viability.
(a) Schematic illustration of the cell viability using the CCK8 assay in HeLa cells. Cells were first treated with 42hb for 24 h, and cell viability was evaluated using the CCK8 assay. (b) Relative cell viability was calculated based on the quantification of absorbance at 450nm. Concentrations ranging from final 20 to 200 nM of 42hb were used. We note that for cell uptake experiments, a final 10 nM 42hb was used. Error bars indicate the standard deviation of three independent measurements; *$p<0.05$, ***$p<0.001$, and ****$p<0.0001$ [t-test].

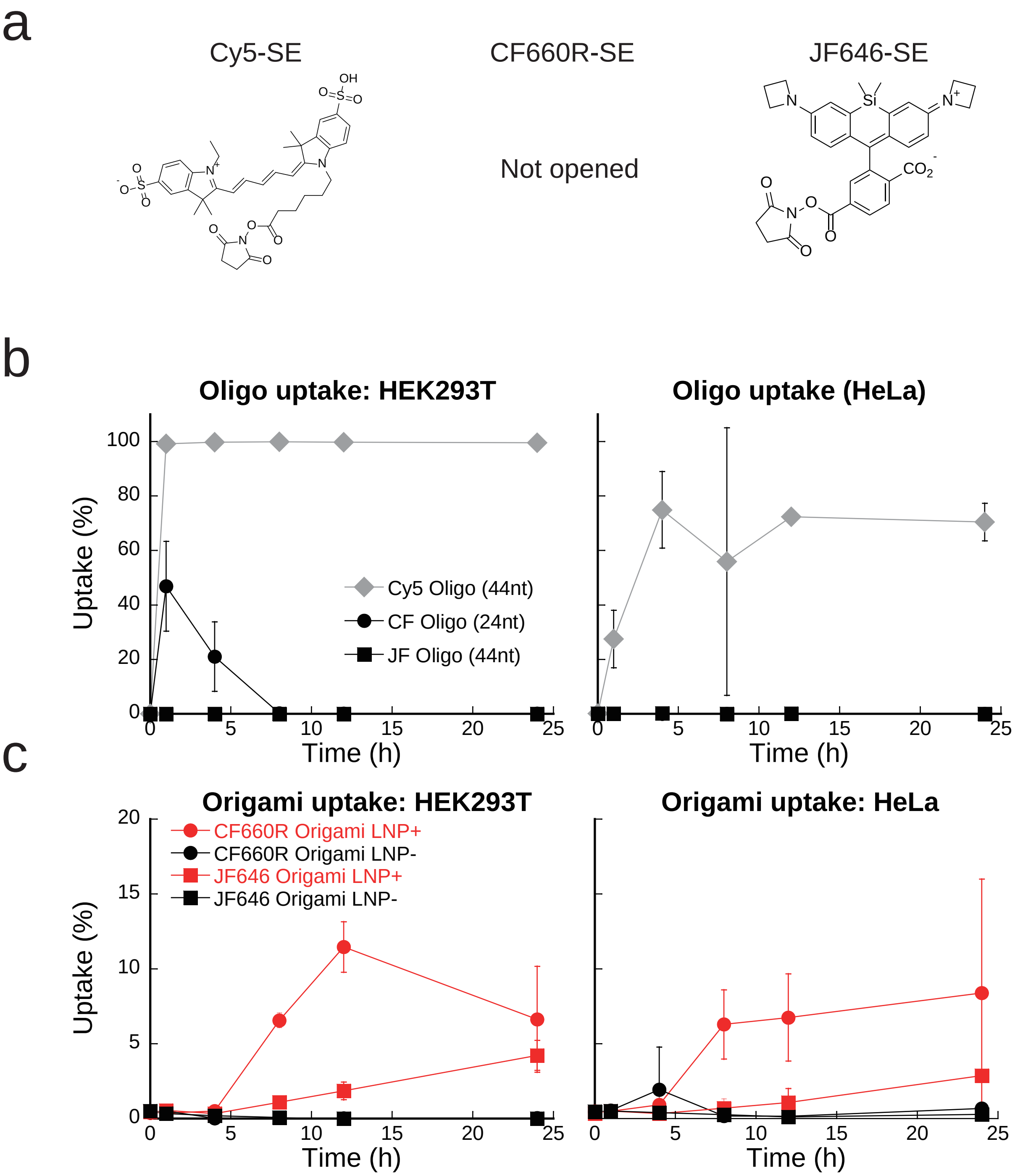

Supplementary Fig. 5. Effect of fluorescent dye on cell uptake.
To evaluate the effect of the fluorescent dye on cell uptake, we switched from the Cyanine-based Cy5 fluorescent dye to Rhodamine-based CF660R and JF646. (a) Structures of Cy5, CF660R, and JF646. We note that the structure of CF660R has not yet been opened due to patent issues. (b) Comparison of oligo uptake by Cy5 (44 nt), CF660R (24 nt), and JF646 (44 nt). (c) Cell uptake of CF660R and JF646 labeled DNA origami (42hb) with and without LNP encapsulation. Error bars indicate the standard deviation of three independent experiments. Final concentrations of oligos and origami were 10 nM in the culture medium.

a

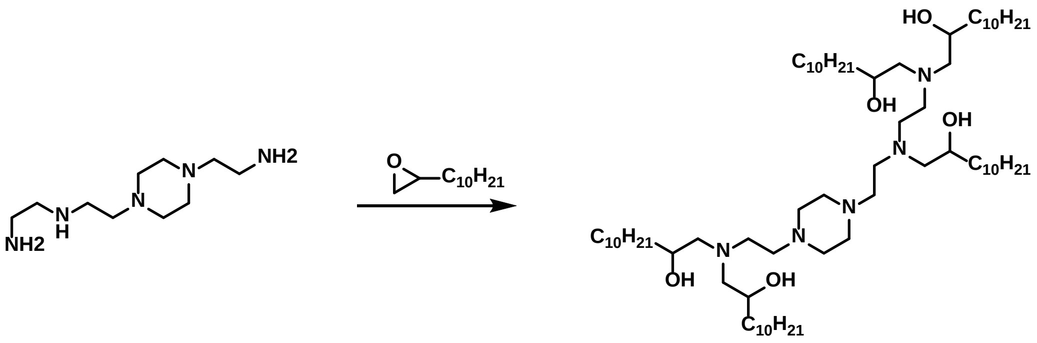

b

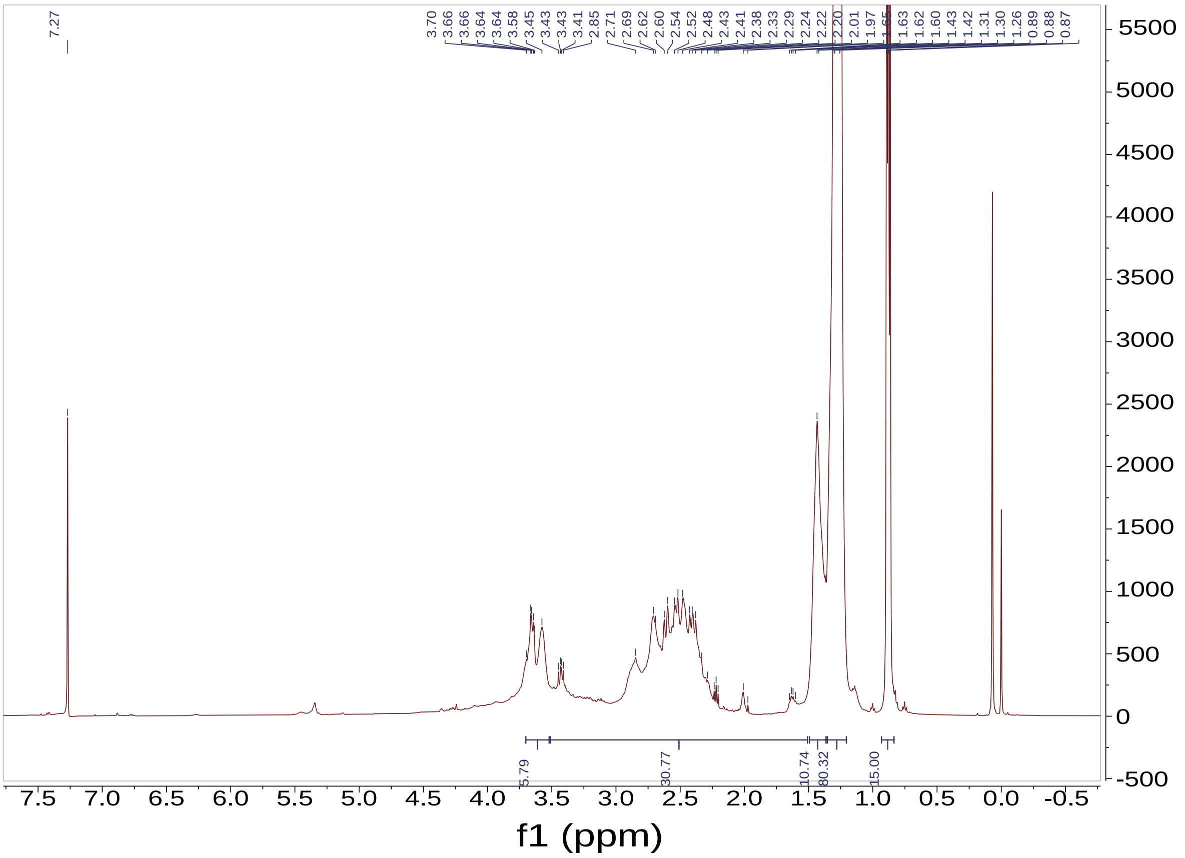

Supplementary Fig. 6. ^{1}H NMR of C12-200.

(a) C12-200 was synthesized based on the previous literature (ref#25, Melamed et al., *J Control Release*. 2022, 341, 206-214. doi: 10.1016/j.jconrel.2021.11.022). The amine N1-(2-(4-(2-aminoethyl)piperazin-1-yl)ethyl)ethane-1,2-diamine (50mg, 1eq) was reacted with the tail 2-decyloxirane (354ul, 7eq) at 90℃ for 3 days to form the lipidoid C12-200. The residue was purified by silica gel column chromatography (Methanol/DCM 1:1 to 3:1) to give C12-200 (200 mg, 76%). (b) The obtained product of C12-200 was determined by ^{1}H NMR in CDCl3: ^{1}H NMR (500 MHz, CDCl3) δ 3.70 – 3.52 (m, 5H), 3.51 – 1.51 (m, 31H), 1.43 (m, 10H), 1.26 (m, 80H), 0.88 (t, J = 6.9 Hz, 15H). m/z: [M+H] + calculated for C70H145N5O5 1137.12; found 1137.093.

a

	Diameter (nm)	PDI	Zeta-poteintial (mV)
UV-	158.3 ±4.3	0.19±0.00	3.2 ±0.7
UV+	150.7 ±1.4	0.20±0.02	3.0 ±0.2

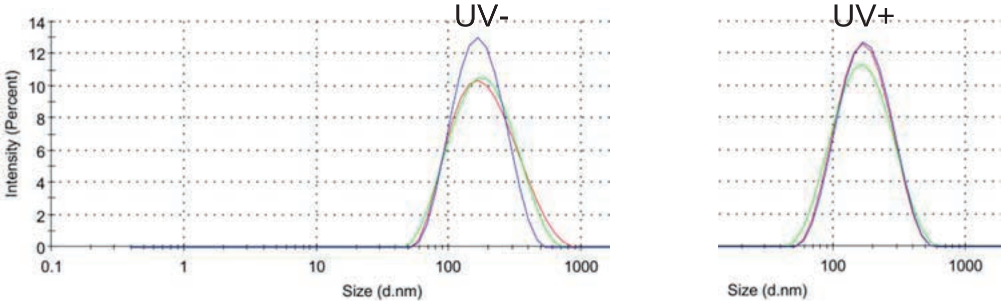

b

	Diameter (nm)	PDI	Zeta-poteintial (mV)
mRNA-LNP	162.7 ±1.1	0.18±0.02	4.4 ±0.3
siRNA-LNP	180.7 ±9.1	0.26±0.03	-3.3 ±1.5
42hb-siRNA-LNP	157.5 ±0.7	0.18±0.00	1.7 ±0.3

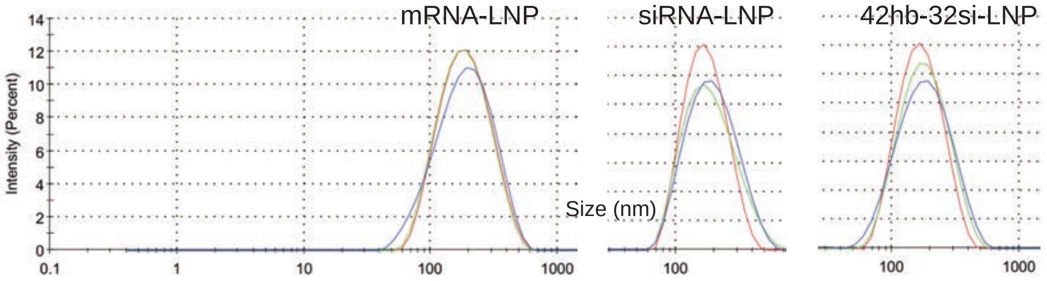

c

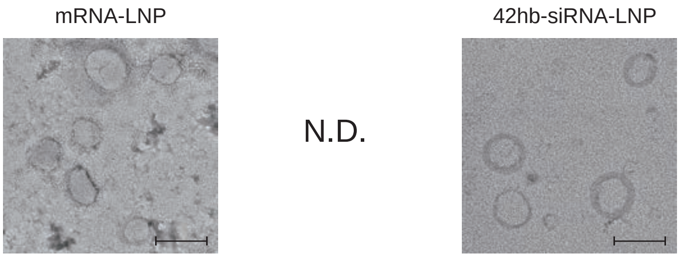

Supplementary Fig. 7. Nanoparticle data.
(a) and (b) Dynamic light scattering (DLS) data of LNPs encapsulating UV+/− (a) and mRNA-alone/siRNA-alone/Origami-siRNA (b). Data are shown in mean ± standard deviation of three independent measurements. (c) Negative staining EM under 120 kV. Scale bar = 200 nm.

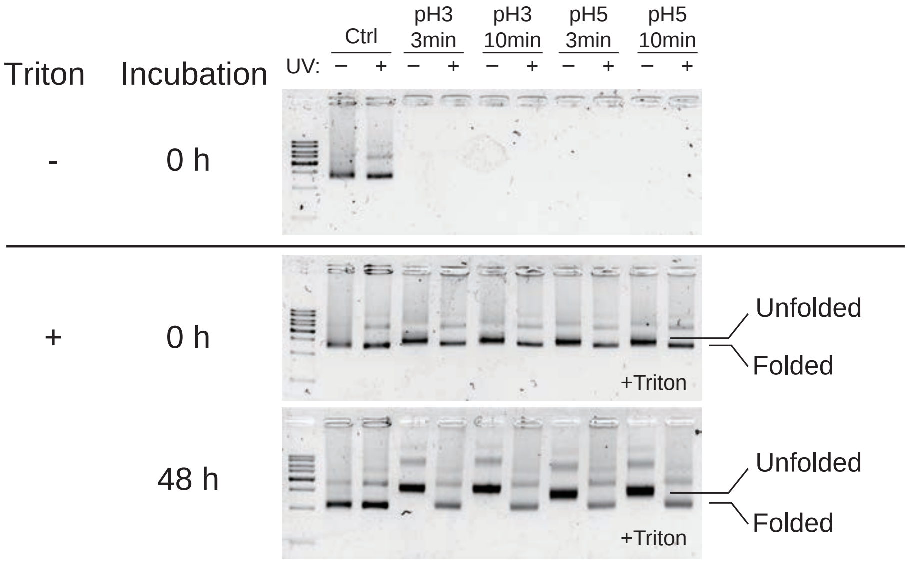

Supplementary Fig. 8. Agarose gel electrophoresis of LNP.
Agarose gel electrophoresis of LNPs before (upper) and after (lower) treatment with triton, a detergent, to break down LNPs. The lower part is the same figure as Figure 4b. Two lines indicate the folded and unfolded 42hb positions. Ctrl, 42hb UV+/− without LNP encapsulation.

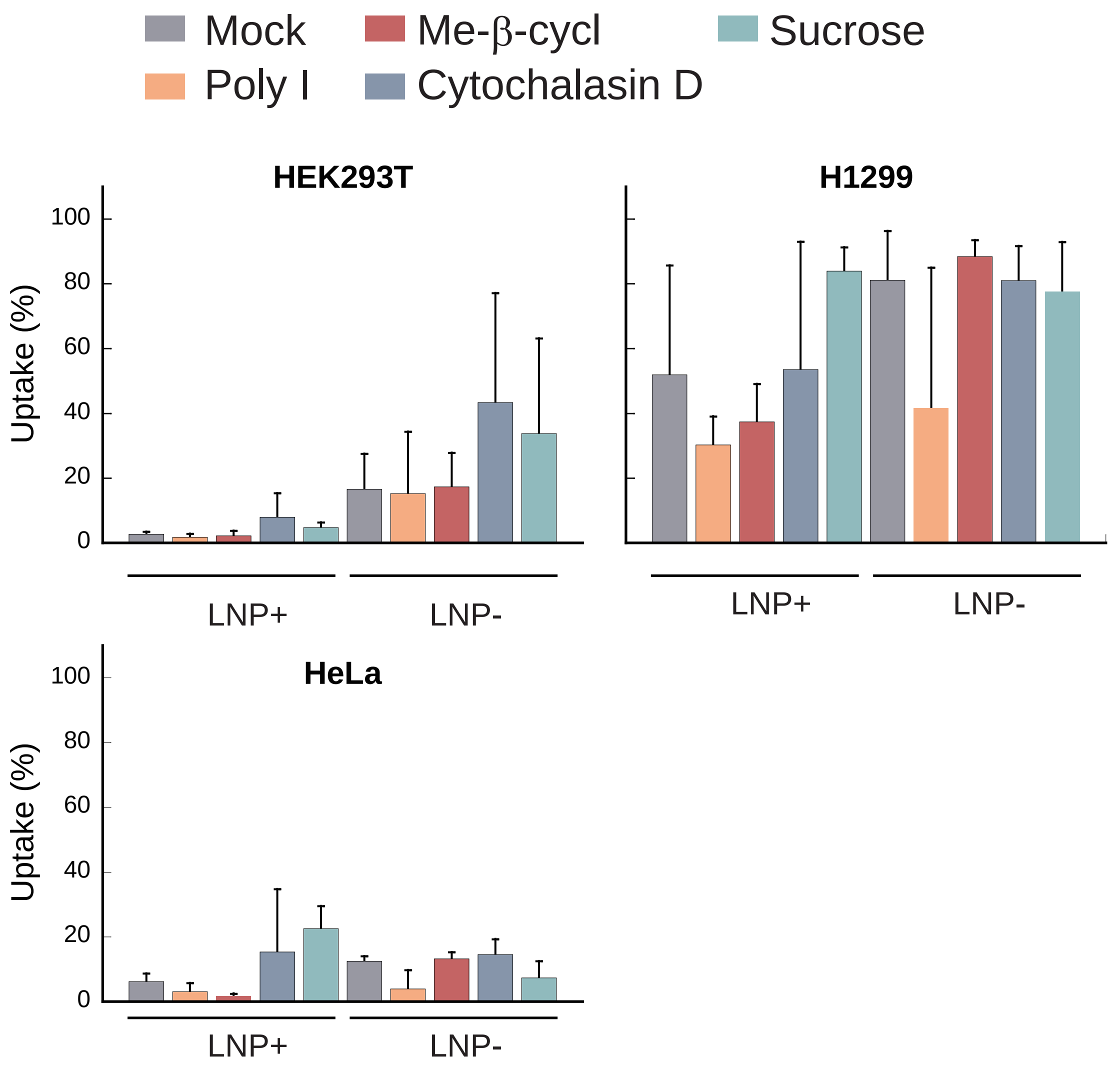

Supplementary Fig. 9. Inhibitor effects on LNP cell uptake.
To identify the cell uptake pathway of LNP, we supplemented inhibitors (Poly I for scavenger receptors, Methyl-beta-cyclodextrin for caveolin-dependent endocytosis, Cytochalasin D for nonreceptor-mediated endocytosis, and Sucrose for clathrin-dependent endocytosis) into the cell culture medium, and cell uptake at 4 h was examined. Samples were infused into the cell culture medium at a final DNA origami concentration ~ 0.21 nM (corresponding to 0.1 µg/well). Error bars indicate the standard deviation of three to five independent experiments.

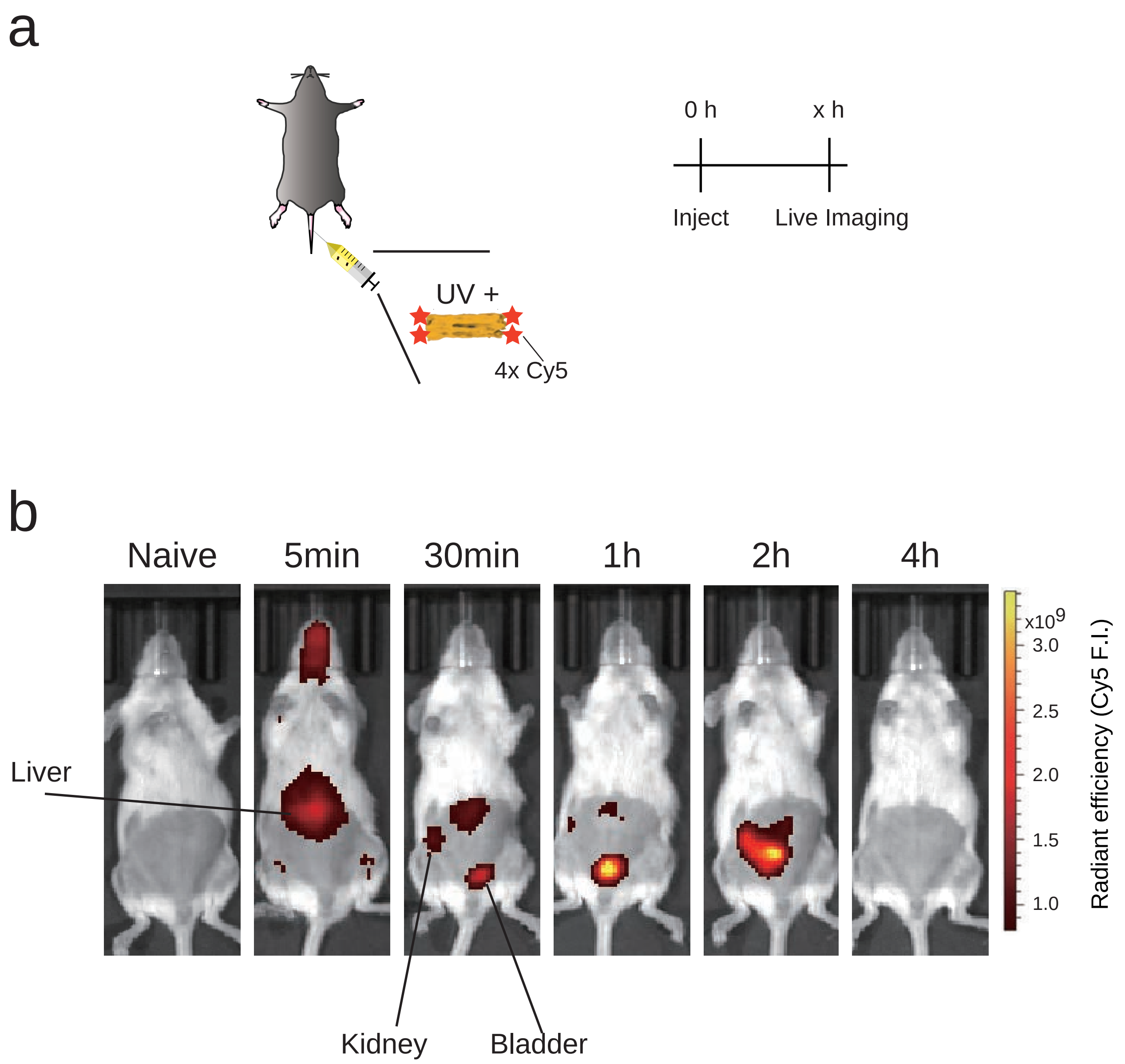

Supplementary Fig. 10. Tail vain injection of naked DNA origami.
(a) Schematic illustration of the experiment examining the circulation of naked DNA origami (unencapsulated by LNP). Cy5-42hb (UV+) was introduced into the mice via tail vein injection at a total dose of 500 nM 200 μL. (b) Live imaging results for up to 4 h after injection. Circulation indicates the complete excretion of 42hb (even UV-crosslinked tough DNA origami) through the liver-kidney-urine within 4 h.

a

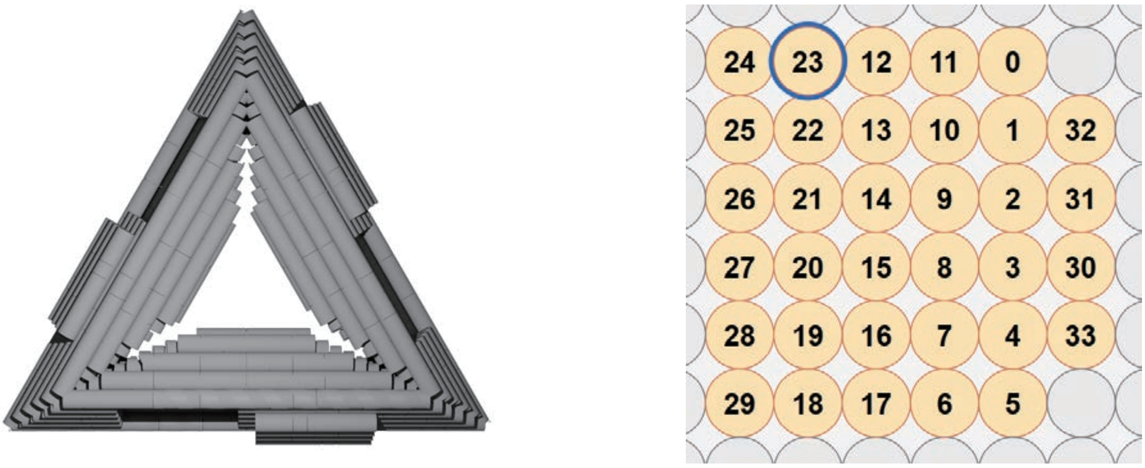

b

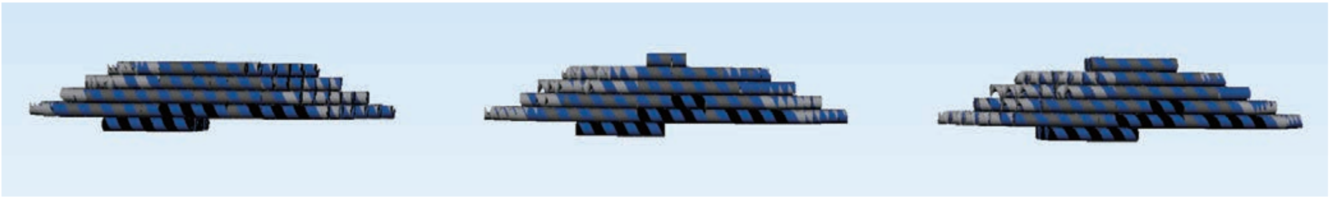

c

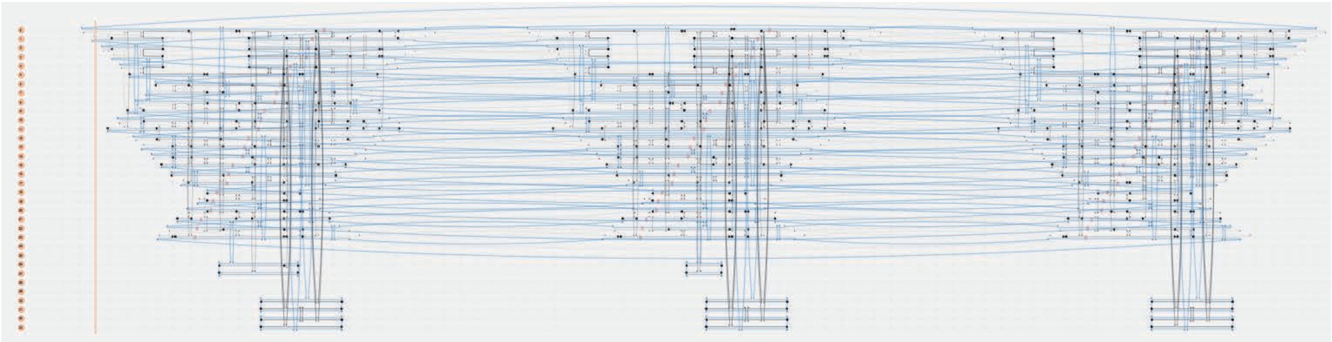

Supplementary Fig. 11. Structure and layout of triangle DNA origami, T1M.
(a) Top view (left, drawn using blender) and cross-sectional image of one arm using caDNAno (right). (b) Maya images of each arm. (C) The secondary structure of the triangle rendered using caDNAno.

a b

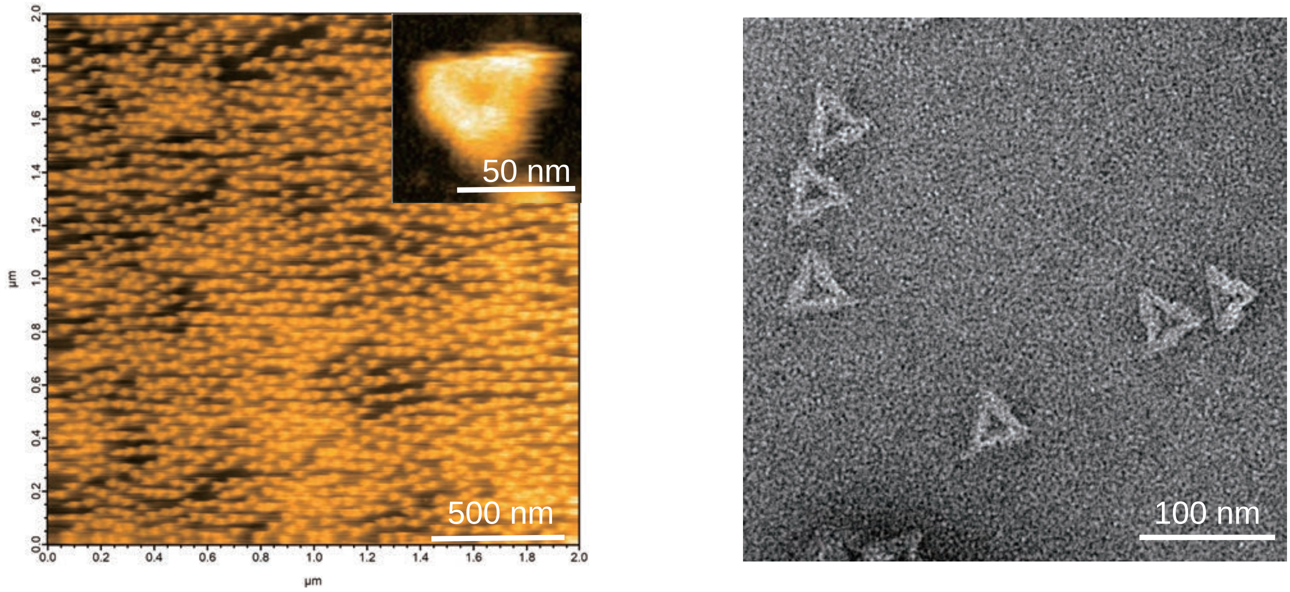

c

	Diameter (nm)	PDI	Zeta-poteintial (mV)
mRNA-LNP	122.2 ±1.9	0.42±0.03	3.3 ±0.7
T1M-LNP	128.9 ±4.1	0.25±0.03	5.2 ±0.5
T1M-mRNA-LNP	116.4 ±1.7	0.24±0.00	7.8 ±0.3

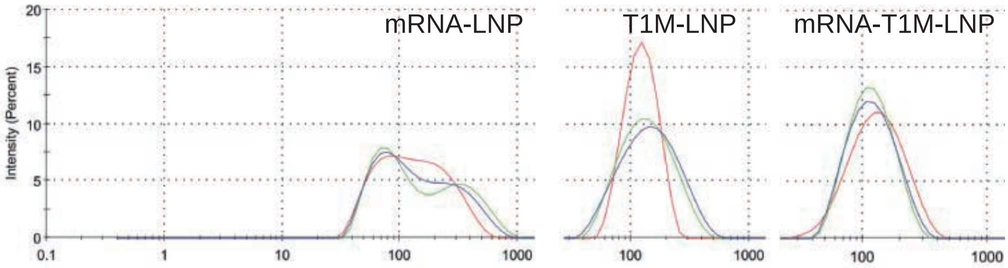

Supplementary Fig. 12. AFM, TEM, and LNP analysis of triangle DNA origami structure, T1M. (a) and (b) AFM (a) and Negative staining EM under 120 kV. (b) Images of T1M. Scale bar 500 nm (wide view) and 50 nm (inset: magnified) in (a), and 100 nm in (b). (c) Dynamic light scattering (DLS) data for LNPs encapsulating mRNA-alone/Origami-alone/Origami-mRNA. Data are shown in mean ± standard deviation of three independent measurements.

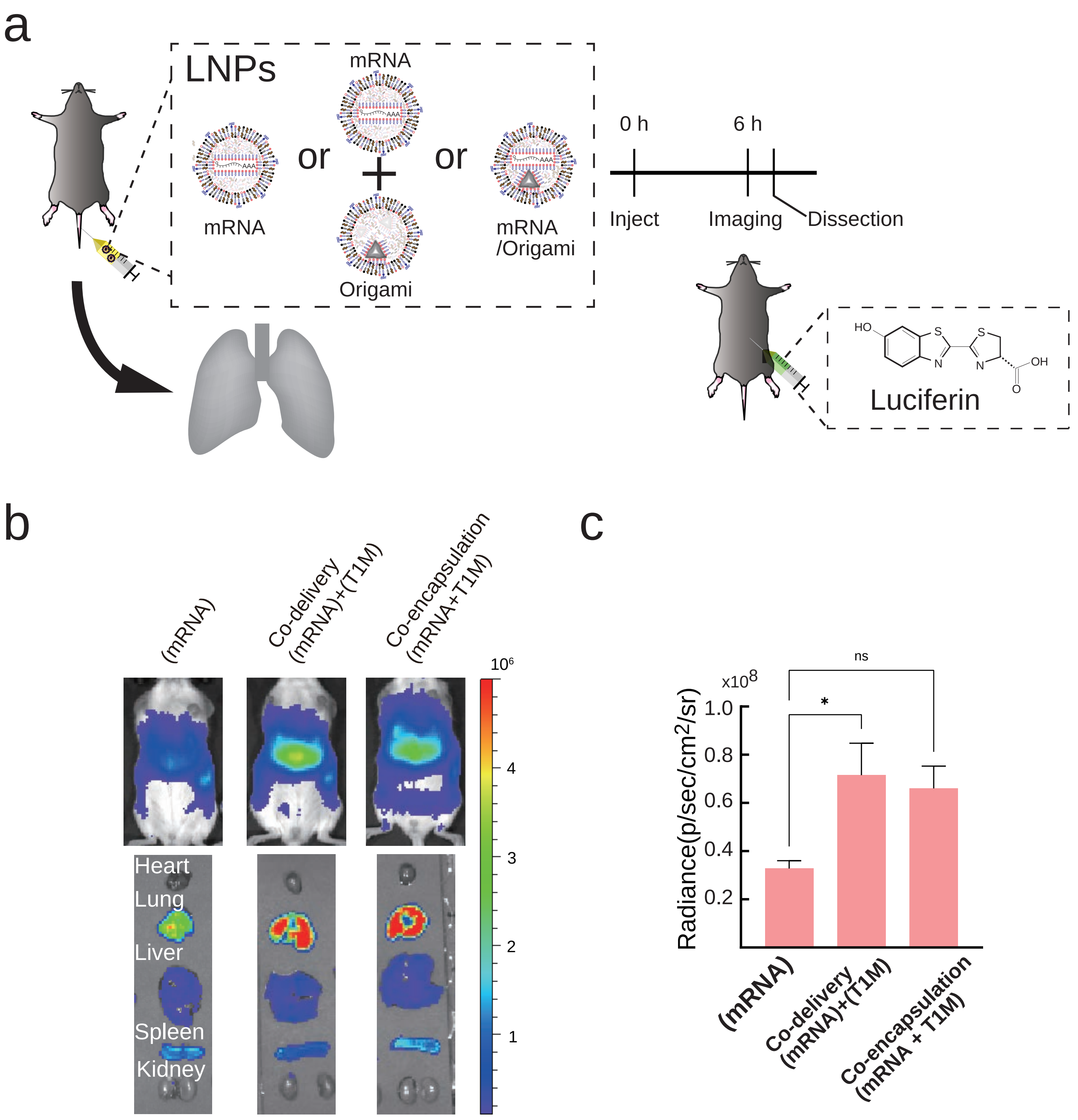

Supplementary Fig. 13. Enhancement of GOI expression in lung by Origami-LNP (Triangle origami, T1M).

(a) Schematic illustration of an experiment examining the positive effect of LNP encapsulated T1M on Luc mRNA expression in lungs. mRNA and DNA origami (UV+) was introduced into mice via tail vein injection at doses of 0.2 and 0.36 mg/kg for mRNA and T1M, respectively. (b) Live images (upper) and images of dissected organs (Heart, Lung, Liver, Spleen, Kidney) after 6 h tail vein injection. (c) Data of dissected lungs 6 h after tail vein injection. Error bars indicate standard error of the mean (SEM) of five independent mice; *p<0.05 [t-test].

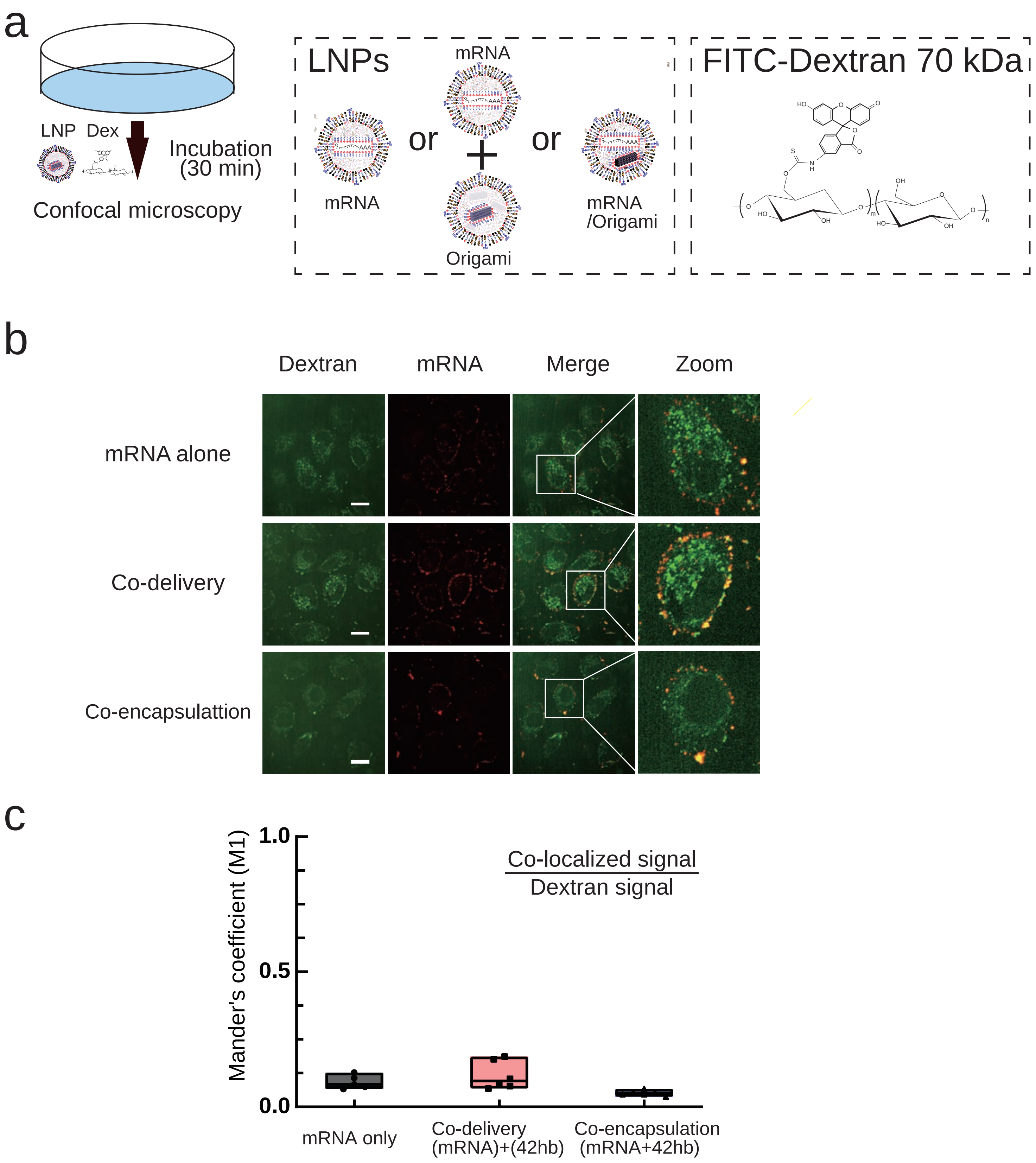

Supplementary Fig. 14. Macropinocytosis pathway analysis (dextran uptake).
(a) Schematic illustration of the experiment examining co-localization of Cy5-mRNA and macropinocytosis marker (FITC-Dextran 70kDa). (b)-(c) Confocal images (b) and Mander's coefficient analysis (c). Co-localization coefficient M1 is calculated based on Dextran. Error bars indicate the standard deviation from six images. Scale bar = 20 μm.

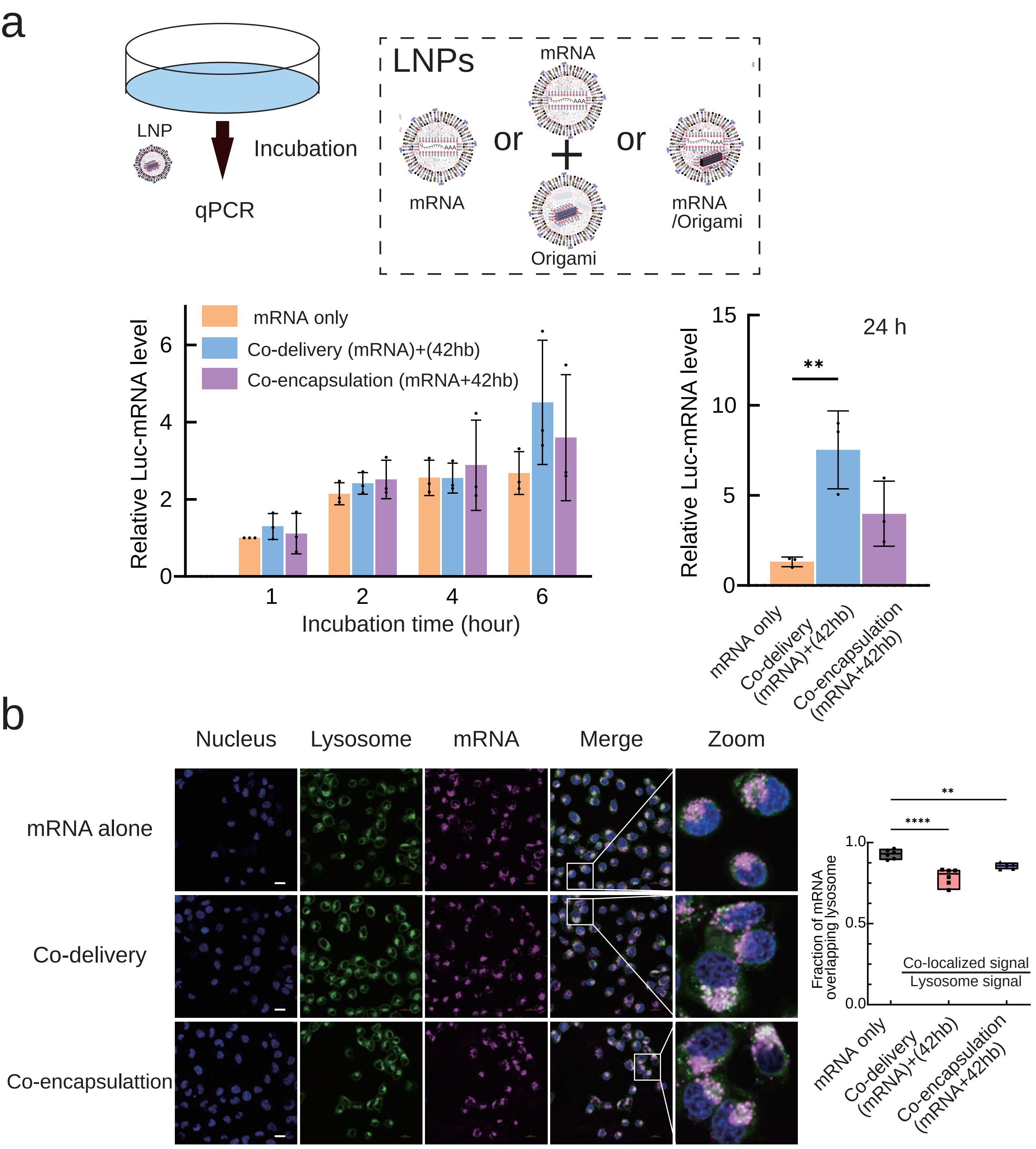

Supplementary Fig. 15. Confirmation of endosomal escape of delivered RNA *in vitro*.
(a) Schematic illustration of the experiment examining mRNA level in cells using qPCR. LNP encapsulated with mRNA alone, co-delivery of mRNA and 42hb, co-encapsulation of mRNA and 42hb were compared. Data from 1–6 h (lower left) and 24 h (lower right) incubation. (b) Lysosomal escape of mRNAs was confirmed using a co-localization assay of Cy5-mRNA and lysosome marker (LysoTracker-Green). Confocal images (left) and Mander's coefficient analysis (right). Co-localization coefficient M2 is calculated based on Cy5-mRNA position. We note that endosome converts to lysosome with maturation. Scale bar = 20 µm. **p<0.01 and ****p<0.0001 [t-test].

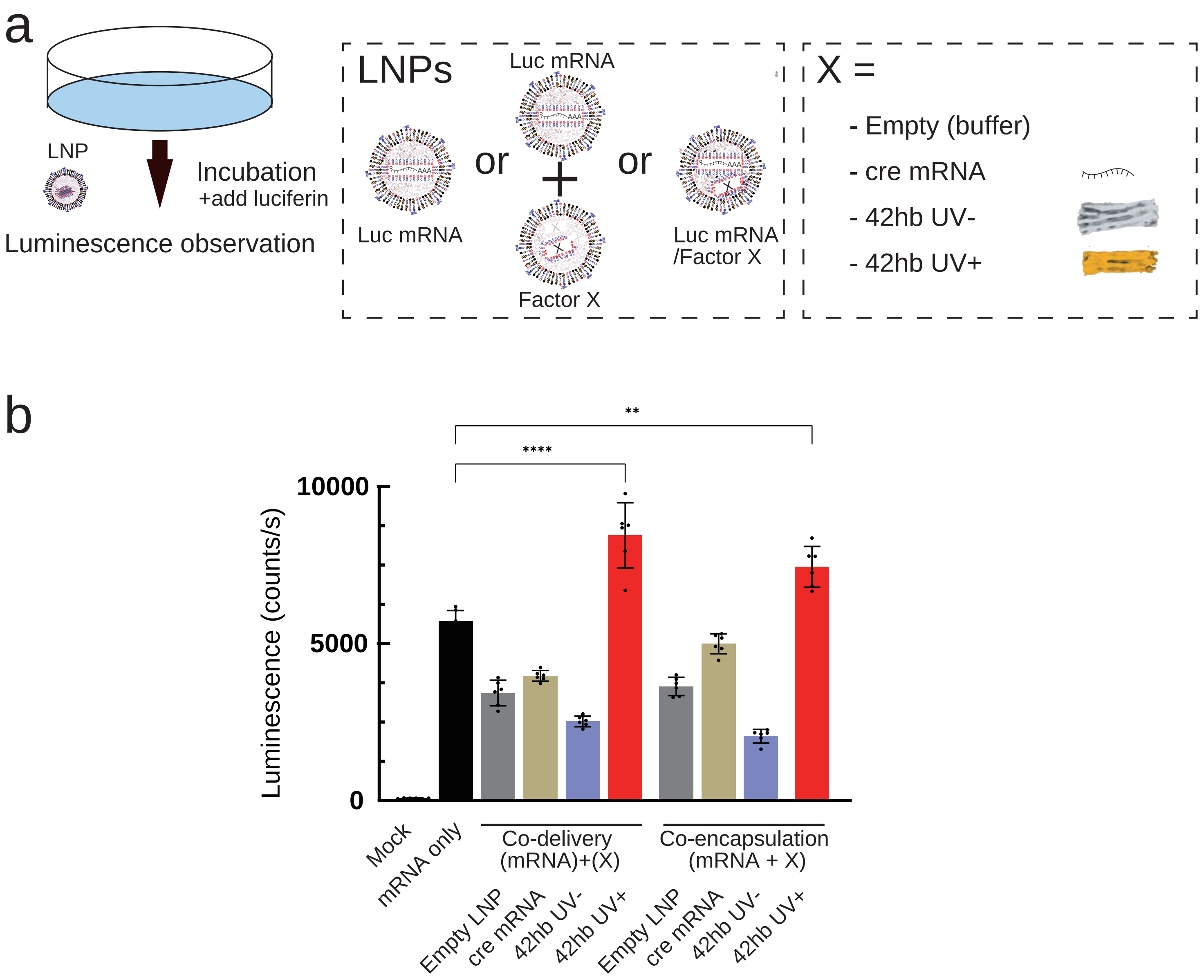

Supplementary Fig. 16. Identification of elements essential for positive effect on GOI function. (a) Schematic illustration of experiment examining the essential element. (b) Luc mRNA was either co-delivered or co-encapsulated with cre-mRNA, 42hb UV-, 42hb UV+ or empty LNPs. The dose of Luc mRNA was 0.1 μg and other nucleic acid (42hb and cre-mRNA) doses were 0.18 μg per well. Error bars indicate the standard deviation of six independent experiments; **$p<0.01$ and ****$p<0.0001$ [t-test].

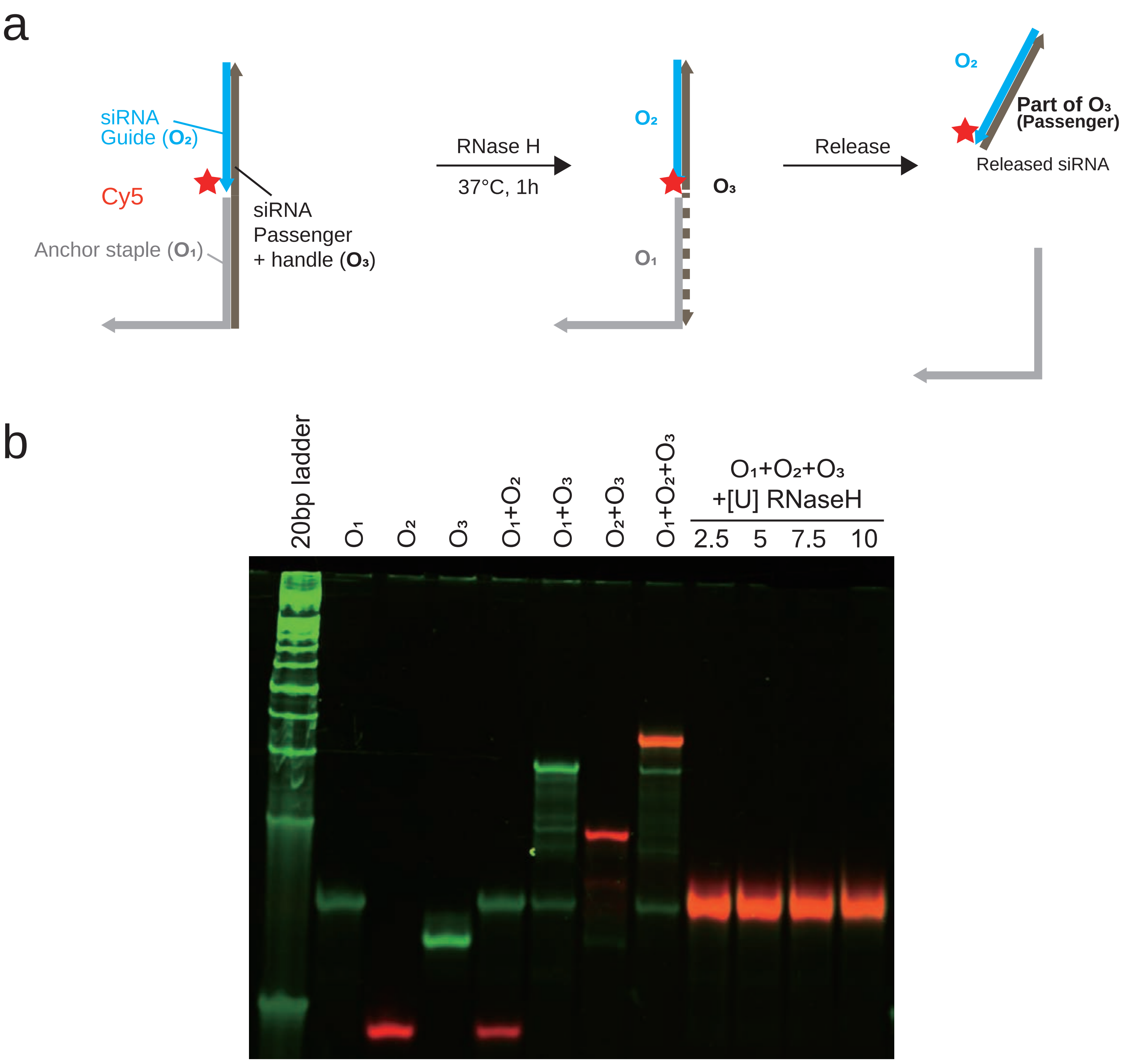

Supplementary Fig. 17. Confirmation of siRNA release by RNase H.
(a) Schematic illustration of the experiment confirming RNaseH digestion of the DNA-RNA hybrid. 3' of guide RNA sequence has Cy5 dye. (b) Results of 15% native gel of RNase H digestion and siRNA release. 1 μM siRNA could be released within 1 h with 2.5 U RNase H.

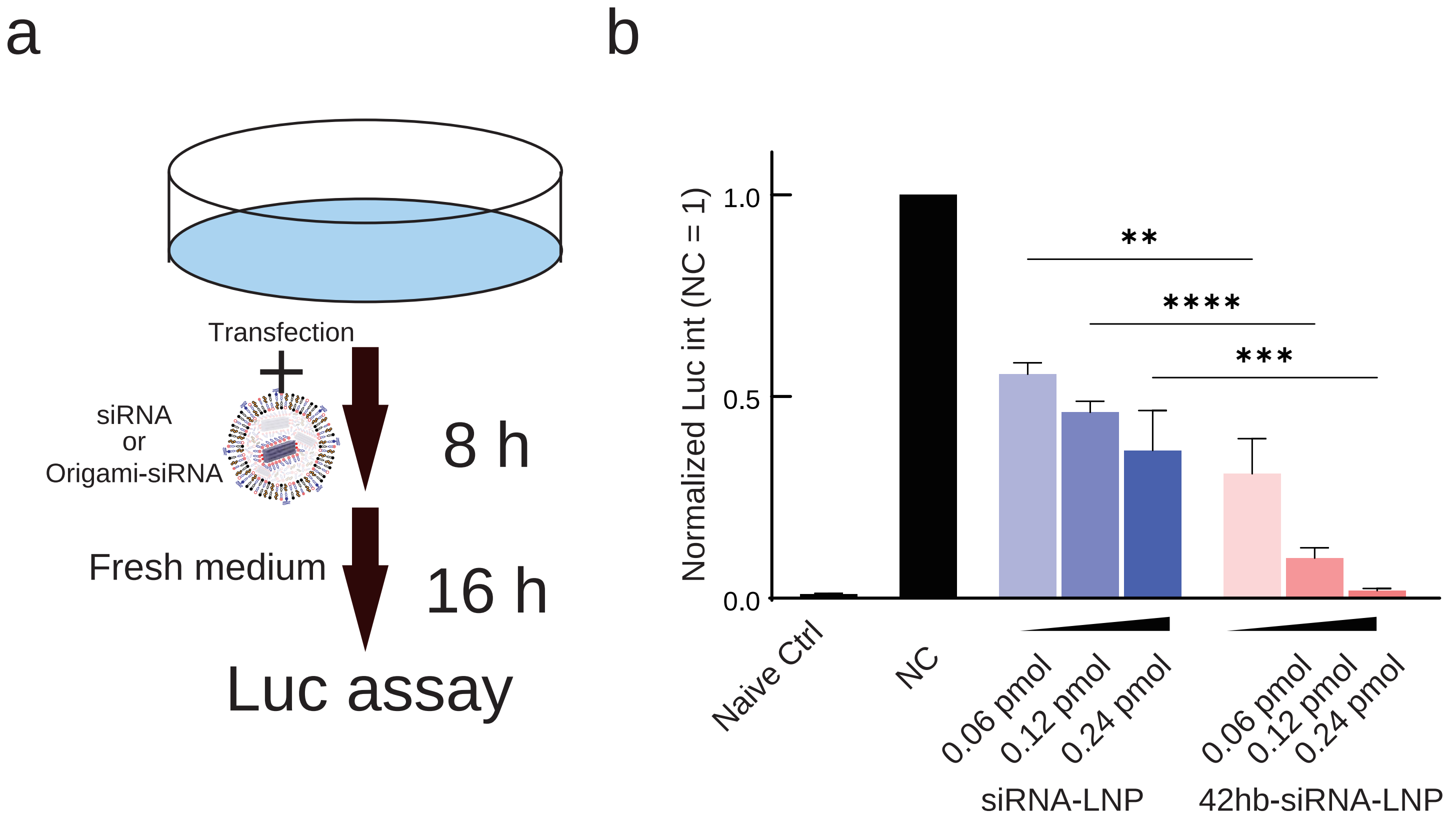

Supplementary Fig. 18. siRNA KD experiment with culture cell.
(a) Schematic illustration of the experiment examining KD effect on target Luc mRNA in HeLa cells. LNP encapsulated with siRNA alone, 42hb-siRNA (same siRNA amount) were compared. The mRNA expression levels and siRNA K.D. efficiency were quantified using the bioluminescence signals.
(b) Relative mRNA levels were quantified by measuring the bioluminescence intensity at 560 nm. 0.24 pmol of 42hb-siRNA mostly KD target Luc mRNA, whereas the KD efficiency of siRNA-alone was not very high. Error bars indicate the standard deviation of three independent measurements; **p<0.01, ***p<0.001, and ****p<0.0001 [t-test].

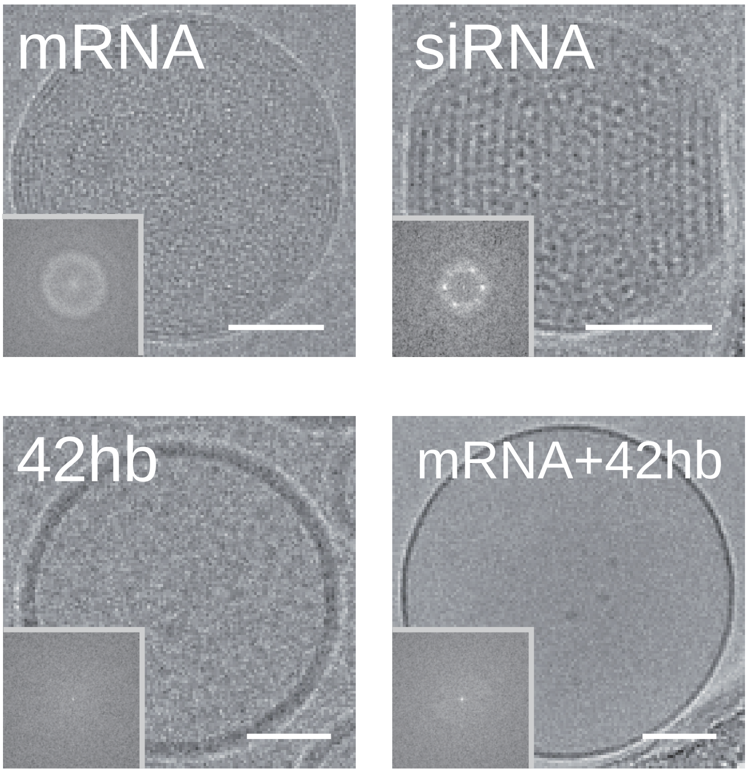

Supplementary Fig. 19. Cryo-EM structure of LNPs.
Cryo-EM analysis of LNPs. Major structures at our experimental conditions are shown. Insets show Fast Fourier transform (FFT) analysis. siRNA-alone LNP showed hexagonal phase (H_{II}) with the lattice spacing of 5.3 ± 0.1 nm (n = 15), Detail structure of mRNA-alone is not clear with current data, but we can observe periodic structure in FFT images with the lattice spacing of 4.8 ± 0.3 nm (n = 32). By contrast, 42hb and co-encapsulation (42hb + mRNA) did not show clear periodic structure with our observed sample. We note that DNA origami-siRNA co-encapsulation LNPs also showed similar tendency (see **Supplementary Figure 20**). Scale bars: 50 nm.

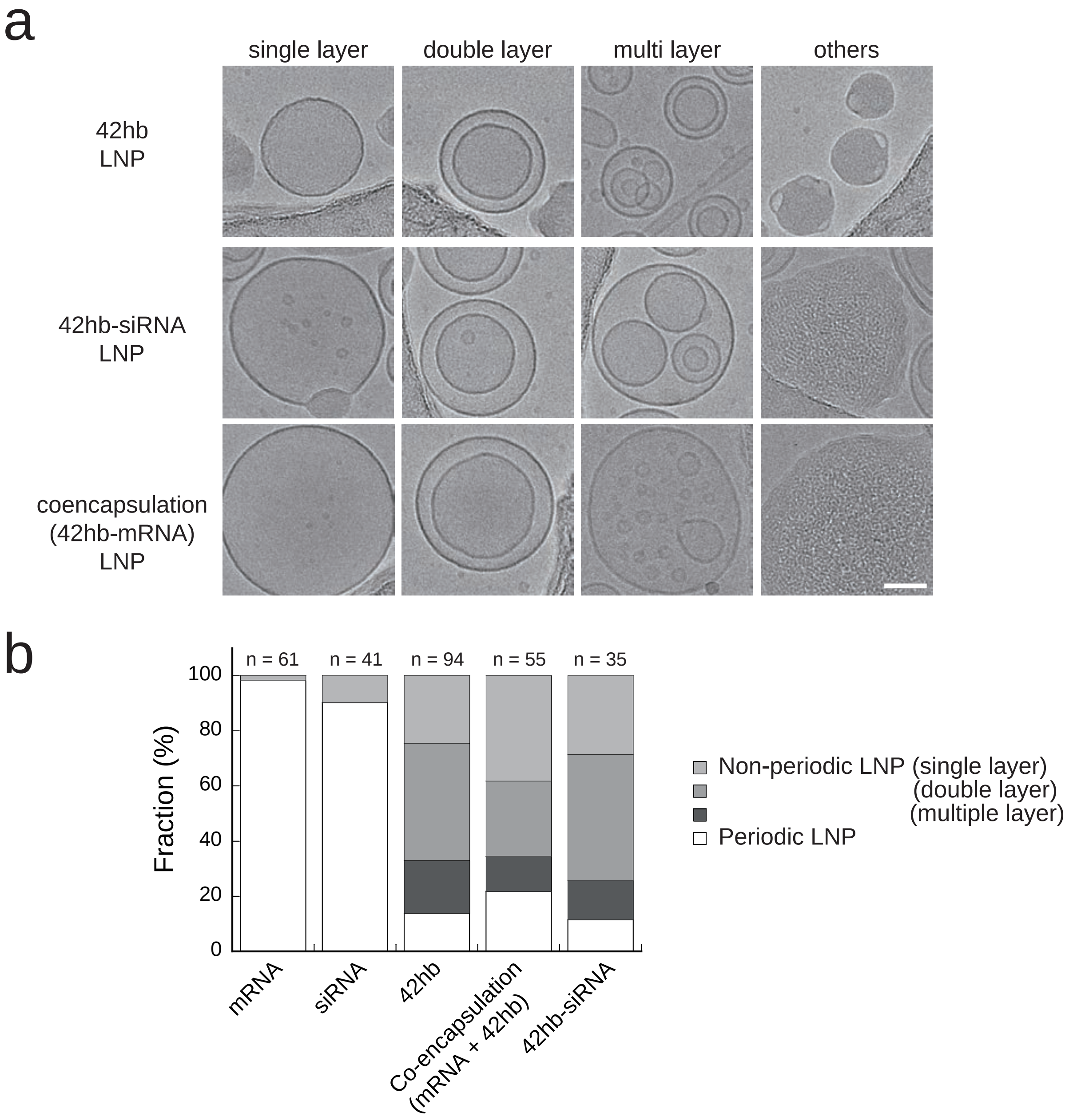

Supplementary Fig. 20. Type of observed LNPs.

(a) Representative Cryo-EM images of DNA origami related LNPs. Scale bar = 50 nm.

(b) Quantification of (a). Data from two independent experiments.

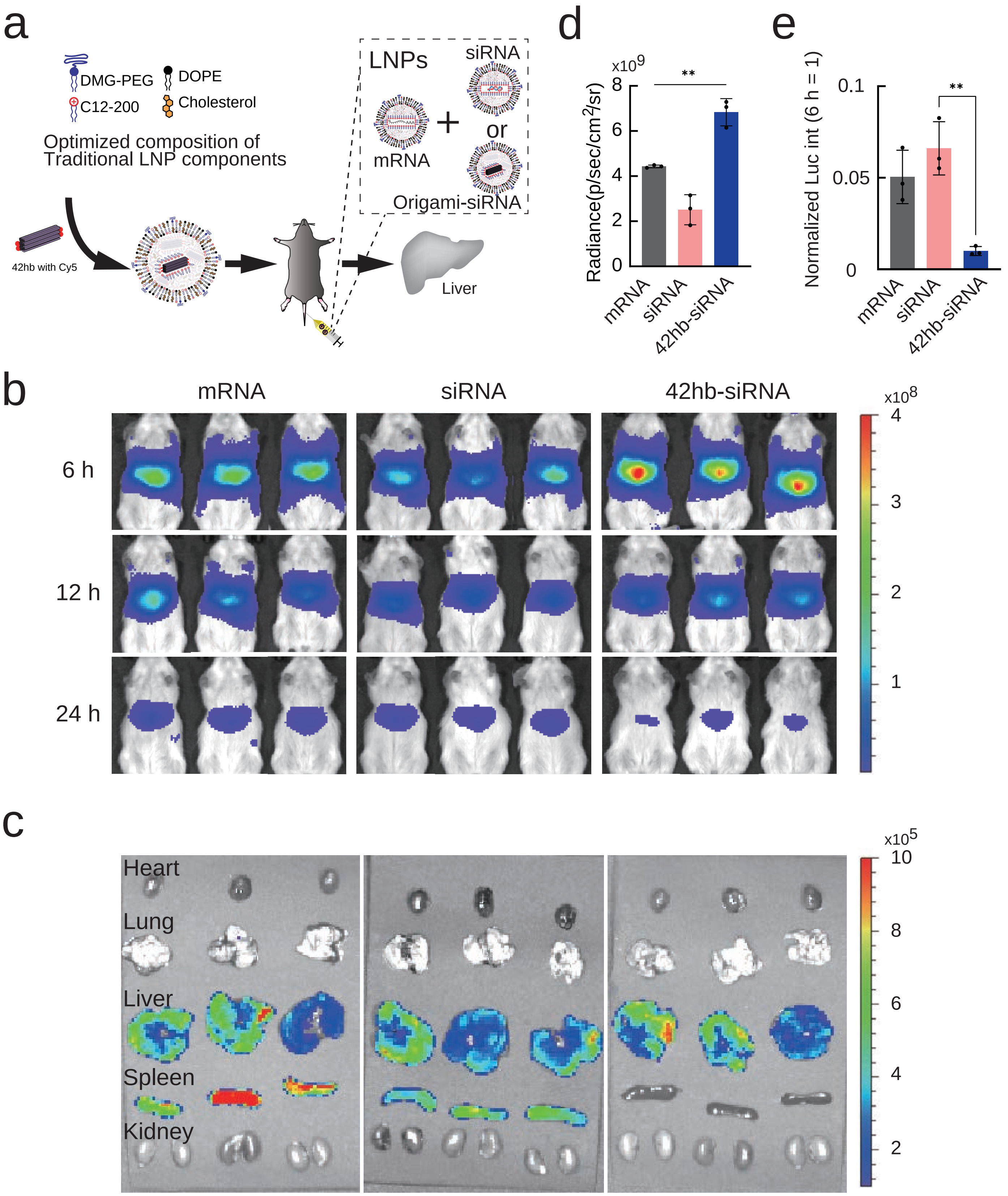

Supplementary Fig. 21. Gene expression control in liver.

(a) Schematic illustration of the experiment examining the effect of DNA origami nanodevices on knock down (KD) efficiency in the liver. We mixed the traditional LNP components (DMGOEG, DOPE, C12-200, and cholesterol). LNP encapsulateing mRNA alone was co-delivered with LNP containing siRNA-alone or with siRNA integrated onto DNA origami nanodevices. The amount of mRNA and siRNA were set to 0.2 and 0.02 mg/kg, respectively. 42hb-siRNA amount was set to 0.36 mg/kg (= 0.02 mg/kg siRNA + 0.34 mg/kg DNA origami). Luciferin was injected intraperitoneally at 6, 12, and 24 h after the first injection and live images were captured 6 min after luciferin injection. After 24 h, the mice were dissected and the organs were imaged 15 min after luciferin injection. (b)–(c) Live (b) and dissected (c) images. (d) Quantification of 6h data of (b). (e) Quantification of (c). Luminescence intensities of 24 h were normalized by those of 6 h for each condition. Error bars indicate the standard deviation of three independent mice. **p<0.01 [t-test].

Supplementary Table 1 | Sequences of staple strands of 42hb.

No.	Sequence (5’ to 3’)
1	TTTTTTAGTTATGATATTTATAAATATTACGTAGATTTACCAGCGCCAAATTAACGAACT
2	TTTTTGCGGTTAACAAACGGCGGATTGATTAAGCAAATAT
3	TTTTTCATCGTTGATGAATATACAGTAACATTATCAATATAT
4	TTTTTAATGTTTCTTTAGGAGCACTAACTTACGAATATAT
5	TTTTTAACTTTGTATCACCGTACTCAGGTTCCGTAATCAT
6	TTTTGCCATTACGACGATTCACTATCATAACCCTTCACATTCTTTTGCCCTT
7	TTTTCAGCGGAGTGAGTTAATCTCCTTAAGACAGTTTTAGACTCCT
8	TTTTAACGTCATTATGGCTTAGAGCTTAATT
9	TTTTAAATTTTACGGAGGATCCCCGGGTTTACCACAGACT
10	TTTGTATCATTAAAGCCAGAATGGAATTACAAATAAT
11	TTTCATAATTTGACGAGAAACACCAGAATTGAAGCCCGAT
12	TTTAGGCATTATATTTATTTAAGAAATTAATAACGTTCGGAAATTATTCATTTCCATTTGT
13	TTTACCCTGATTAATCCCCCTCAAATGCTTTGGAGACAGT
14	TTTACAGGTATTATAGAAAATTCATATGGTTTAAATACATAT
15	TTTAATGCCCCCTGCCTATTTCGGAATTTTGCTCAGTAT
16	TTTAATCATTGCGCGTTTTAACCGCCTTCTCCATGTTACTTATTGCGATTAT
17	TTTAAGAGTTCGAGTAGTTTCATCAGTTAGGAGCCTTAAACAACTTTCAACAGT
18	TTTAAAGGCCTTGTATCGGTTTATCAGCTTTTACAACATTAT
19	TTGTGAGTGATTTTGAAAGGAATTGAGGAAT
20	TTGCTGAATTTTTTCATTTGGGGCGCGTTAGCATGTAT
21	TTGATGAAATTGAAGTATTAGACTTTACTTCTGGAGTGAT
22	TTGAGGCGGTCAGTATTGCCAGCATTAACCTCATTTGGCAAATTCTGATTAT
23	TTGAGAAGTGTTTTTATTTTGCCCTTTTACTCGTCTTCAGGGCTTTAAATTTTT
24	TTGAATGGCTATTAGTTTTACCTCTTAGTGCGGTTCATTTGATTTTTGCCCT
25	TTCTGTATGGGATTTTGCTTTTTTAATTTTGCTTTTGT
26	TTCTGGAGCAAACAAGATTGGTCATTGCCTGAGAGT
27	TTCGTCTTTCCAGACGTTTAGGTGAATTCGGTCGCTTCCGTCGAT
28	TTCGAATTTTCAGGAAGTTGTCAATCTTTGAACGGTAATCGTTTAGAGATCTTTATGACCT
29	TTCCAGACGATTGTAGGTAAAACATGTAATTTATAGCTTT
30	TTCATATGCGTTATACAAATTTTGGGTTAT
31	TTCAGGCTGCTTGCCAGGGTTTTCCCAGTCTTGAACGTTATT
32	TTCACTGCCCGCTTTCCATTCTGAAAGT
33	TTCAAGAGAAGGATTATTTGCCATCTTT

No.	Sequence (5' to 3')
34	TTCAAATCACTTATCATAATTACTAGAAATTCAAATCCAT
35	TTCAAAAATTCGATTTTTTGGGAAGATTTACCGATTTCGATCTAAAGTTTTGT
36	TTCAAAAAAGACGCTGAGATTAAAAATCTAAAATCATTTT
37	TTATTTTTGTTAATTAATGGTTTGAAATTTTCTTCTGACT
38	TTATAACTATATGTAAATTTAACGCCAT
39	TTAGATAATATTCCCTGCCATCTGTAAGCATTCACCGCCTGT
40	TTAATTTTAATTACGAGCACGTATAACGTGTTCCAGAATCCT
41	TTAATTGCTTCCCAGCATTTCAGGCCGATTAAAGGGT
42	TTAATGCAGTTATATATTTTAAATGCAATTCTGTAGCCAT
43	TTAAGTTTATTGAAACGCAAAGACACGTTTACCAGT
44	TTAAGAATTAGCAAATTCCAACAGTTGCGTTTTTTATAAGGCTTAACTAATT
45	TTAAACAGTTTACCAAGCGCGAAACAAAGTACAATTTCGAAATCCT
46	TTAAACAGGAATTACTATTAAAGAACGTGGT
47	TGTTTTTTTTCCATCATTCCGCGCTTTCGGGGAAAGCCGGCGAACT
48	TGTTTGCCTTTAGCCCGTTTCGCCCACGCATAACCGATATATTTTTTTCTTAAAT
49	TGTTAAATAATTAAACTTTTTCAAATATATTTTAGTTTTTTAATATT
50	TGTGGCGAGAAAGGATTACGCTGCTTCATATTCTTTCAACAGTTATAACCTTTCATAGCGT
51	TGTAGCGACATTGGCTCATTAAAATCACCATTTAGCTATCTTACGAATGAT
52	TGGTTTAATTTAGCAAAGCGGATTGCATCTTCAATACTGCT
53	TGGTGTAATTTTCGCCATTAAAAATACCTTCACACGACCT
54	TGGTCAATAATTCTTTCCTTATCATTCCAATTTTTGAATACT
55	TGGGGCCTTTTCCAGTGAGACGGGCAACTTCCAACGCT
56	TGGGCGCGTATTGAGGCCACCGAGTAAAAGAGTCTGTTCTTTTCATTGAATCGGT
57	TGGGCGCAGAACAATATTACCGCCAGTTCGTCTGAT
58	TGGCCTTCTTTGCCTGATTAAAGGCCTTTTAAACATTTAAGACTTTCAACCGATTGAGGGT
59	TGGCAAAATTGGAACCGAACTGACCAATTATCACCGGT
60	TGGATTCTTTCAAGAAATTAAATCGCTTAAGCGCCATTCGCCATT
61	TGGATTAGTTCGGGATCGTCACCCTCAGCAGCGATTAAAAAAAAGT
62	TGGATAAAAATTAGCGAGTAACAACCCGTCTTTTTGTTAAAT
63	TGGACGTTTTAAGAACTTTGAATCAATTGCCGCCATTCCTGATAAATTGTGT
64	TGGAATCGTCTTTCAACCGTTCTAGCTGATTTAAATAAGGCT
65	TGCTTTCATTTTCAGCTCATTTTTTAACTTCTCCTGGTTT
66	TGCTGGCGAAAGGGGTTCTCTTCGTTTTACATCTTCGAGAACTTAAACAACTTAACAAAGT
67	TGCTCCAAATTTTGAGATTTAGGAATACTTTCCACGGAAT

No.	Sequence (5' to 3')
68	TGCGGGGAGAGGCGGTTTTTCACCAGTT
69	TGCGACCTGTTACCCTCAGAGCCACCACTTTTCCAGAGCT
70	TGCGACATTTTCCTTATTACGCAGTATGTTAGCAATTTTCATTGT
71	TGCCCTGAGATTAACCTGTCGTGCCAGCTGCATTAATT
72	TGCCCGTATTGAGGGTTGATATAAGTATTTTTAGCGTT
73	TGCCCAATTTGAGCCAGCATACCAGTCATTAGGGAAGGTAAATATTGAT
74	TGCAGCCTTTTAATATCCCATCCTAATTTTCTGTAATACT
75	TGCACTCATTTGGGAGAAACAATAACGGTTAATTACCTTT
76	TGCAAGGCGATTTTATCATTTTGCGGAACTTCCGCTACAT
77	TGAGGGGGTTAAATATCTTGTCAAAAATGAAAATAT
78	TGAGCCGCCATTAACAGCCATATTATTTATTTCATAAAAACT
79	TGACCCCCATTGCCGGAACGAGGCGCAGATTCCTCCCTCAT
80	TGAATACCCAAAAGAACTGGCATGATTTGTTCAGAT
81	TGAATACATTTTCTGAAACATGAAAGTATTAT
82	TGAACGAACCACCAGTTCTCCGAATTCTGACGCTTGGTTATCTTCCACCAGT
83	TGAAAGATTTTAAATTGTTCCCTTATTTGGAACCGTTCGGTCAATCATAAGT
84	TGAAACCAATTTCGAATTATTCATTTT
85	TCTTTGAAAGAGGACTTAAGGCACTTTGAGGAAT
86	TCTTGCTGTTGCAAATGTTAGAGTCATTGCTTAGGTTTTCTTACCAGTATAT
87	TCTTACCAACTTGTAATTGAGCGCTAATATTTGTAATTCTGT
88	TCTCTGACTTCAATAGGTTGCTGATGTTAAGCCTGTTTAGTATTGGGTGAGTTGTAATGTT
89	TCTCTATGATTGGTCCACGCTGGTTTGCTTGTTGCGCT
90	TCTCACATTTGGAAGCATTCACAATTCCACACATTACCGAGCTTGCTCGCCT
91	TCTATTATTTCGGGAGATTCCAGTTATTAAGTACGGTGTCTGGAT
92	TCTATTACGCAGGGTTAGAACCTACCTTACTTCTGT
93	TCTAATTTGTTATTAACTGAACACCCTGTTATGTTCAGCT
94	TCTAAATAACTAGCATTTATTGTATTTCCGTAATTTTTTGAGCAAAAGAAGAT
95	TCGGTACGTTCTTTCCTTTGGGCGAAAAACCGTCTATT
96	TCGGGGTTTTCCTATTATTCTAAAACACTCATCTTTT
97	TCGGAGATTTGTGCCTTGAGTAACAGTT
98	TCGCGAGATTGAATAAACACCGGATTCATCAATTTAACCCTCT
99	TCGCGAACTGTTCCAACAGAGATAGAACCCTTCTGACTTGTCGGGATTGAGTTGCT
100	TCGACAATTTAAGCAAGTTCCCAATTCTGCGAATTTCCATATTTAGCGTCTTTCCTCAGAT
101	TCCTTCATTTAGAGTACCTTTAATTGCTCT

No.	Sequence (5' to 3')
102	TCCTGTTTAGCTATATTTATAATGTTCAAATAATTCACCACCTTTAGCGTTT
103	TCCGTGGGTTGAGAAGCTTTGCCGGATTGACTGGATTTATAAAATTTTTTGTCACAATCAT
104	TCCGGAAATTCGACGGCCAGTGCCAAGTTAATTCGACT
105	TCCCTCAGTTTTCATCGTTATAAGTGTTTGAGGCTTGCAGGGAGT
106	TCCCAATCTTCTGTAGCTCAACATGTTTTAAATATTTTTCGCAAATT
107	TCCATAAATTTCAGAGGTTGCTAACGTTAACAGTTGATTTTAGCGAT
108	TCCAGGCGGTTGCATTTTCGGTCATAGCCTTGGCTTGAGATT
109	TCCACACCCGTTCGCAAATTAACCGTTGTAGCAATACTTGGCCTTGT
110	TCATTAGATTAGTCAGATTTTCAACTTTTAACGGATTGCTTTCGTTTAGTAAATGAATTTT
111	TCATTACCTTCATCGATTTCAGCATTTTCTTGATATTCACAAT
112	TCATGACAAGAACCGTTAGACTTTTTCATCGGAT
113	TCATAAAGGTGGCAACATTTAGCGTCTTAAAAAGATTAGAATAAT
114	TCAGCTTGATTAAAATCTACGTTAATAATTGACAAAAGGT
115	TCAGACTGTATTTTGTGAATTACCTTATGTTTCAGGTCTT
116	TCAGAAGATAAAACAGAGGTTAATGGATTT
117	TCAATTTTTTCCGCCGCTTAGCAGCATTAGGTTTATTCACCCTCATTTTCT
118	TCAATTACCTGTTCGGCACCGCTTCTGGTGT
119	TCAAGTTACATTACAAAATTAATTACATTTTTAGCCGTCAAT
120	TCAACTAAATCGGAATTTGCTTTGTTAAGTTTGTTAGATTAGTTAACAATTTTAAATGTGT
121	TATTTTAGACAGGAATTAGCAAGCTTTACCGACTTGATAAAGTTGTAAACGTTAATTTCAT
122	TATTTCACATAAGCATCACTTCTGGTAATATCCCAGGGTGT
123	TATTTAGAGCTTGATTTAATGCGTTAAAGAAATTTAAAATATTGAAACAGT
124	TATTTACATTGGCAGATTTTGCGTATTT
125	TATTGTTTGGATTATTTATATCAATTAACGTCATTTAGGAATTTAGGTAAATTCAGAGAGT
126	TATTCTGGTTATAGCCCTAAAACATTGAGTAAATTGGTGGGCTTAACTAATT
127	TATTCGCCTTTTGGGAAGGGCGATCGGTGCGGGCTTGATGTGCTT
128	TATTCGCATTTTTAAGCTACGTGGTGCTTGTTTCTTTAATGT
129	TATCGCAAGACAAAGAATTTGTTAAATTCAACATTTTTCATTTGT
130	TATCCTCATCGTTCCAGAACCACCACCAGAGTTATCCTGAATT
131	TATAGCAATTGTAGCACTTGGAATTATTAGGAACCTTCATTCCACAGACAGCCCTCATT
132	TAGTTTCATTTCGAGTAGATTTAGTTTGTTCCAAGTACCT
133	TAGTTAGCGTAATTAGTTGCGCCGACAAGCAAT
134	TAGTAATAAAAGGGACTTGAATCGGTTAGCTGATT
135	TAGTAACATTTTAAGTTGGGTAACTTGCAACTGTTTGATTGCTTGAACGGGTTCTGTTTAT

No.	Sequence (5' to 3')
136	TAGGGATATGACAACAACCATTGAATAGGTTGGGGTCAT
137	TAGGGAAGCGTTTCAACAATAGATAAGTCCTTTCAACGCAAT
138	TAGGAGAATTAGCTTTCTTAGGGGACTTCAGGCAAGGCAACGT
139	TAGCTGAAAAGGTGGTTTTTGCGGTTGGATTAGTTCAAGAGTAATCTAAT
140	TAGCGCAGTTGAGTGTACTGGTAATAAGT
141	TAGATTATAATTCGCGTCTTTTACATAATTGTACCTTT
142	TAGAGTCCTTAAGAATATTCTTAAGTGTCCTACTTCTTTCTCT
143	TAGAGGCTGCATTCAACCTAAAACGAAAGAT
144	TACGACGTTGTAAAATTCCAGGCATTGCAGAGGTTCAATAATTTGAAAAATTTACAGAGT
145	TACCTCCCTTCTAAGAACGCGAGGCGTTTTCCGTTTTTAT
146	TACCGACCGTGTGATTTAAATTAATTCTTTATTTTTGAACAATTCGGCTGTT
147	TAATCGCCTTGAGGCATTTCGACAAATTCATTACCTTGCTTATCCGGTATTTTGACTTGCT
148	TACTCCAACGTCAAATTCGTTAGATTTAAATCCTTGGATTTATTCAAACATT
149	TAATCAGTTTCTATGGTTTCCCTAAAGGGAGCCCCCGT
150	TAATATTTTTTCGTAAGAATACGTGGATACGAGCCT
151	TAATAGTGCTGAATTTTCTGCCAGTTGGGATAGTTAAAACATTTTACAAAGT
152	TAATAATGGACATTAAGGAGCGGAATTATCATTTGCGTAACCAT
153	TAAGACTTCTTTAATAGTAAAATGTTTATTGAGGGTAGCT
154	TAACTCGTATTTATCAGAGCGGGAGCT
155	TAACGCGCTTTATTAAATTACCATTAGATACATTTGCAACTATTCAAAATAT
156	TAACCAGAGCTTGAAACGATTTTTTGT
157	TAAACGACGAAGCCCTTTTTTACAACGCCAGATTCAAAAT
158	TTTCTTTGTTAGCGGTCTTAGGGAAGAAAGCGAAAGGAGCGGGCGTTTTTTTTT
159	TTGTACCATTGTCACGTTGGTGTAGATGGTTTTTTTTT
160	TTGCTTCTTTCAGGTTTTTAATTATTTGCACGTAAAACAGAAATATTTTTTTTT
161	TTCTAATCTTATCCTGTTTGATGGTGGTTTTTTTTTTT
162	TTCAGTGATTAATTCGAGCTTCAAAGCGATTTTTTTTT
163	TGTTTCCATTAAACGGGTAATTTTTTTTTT
164	TGTACCGCTTGATACAGTTTCTCTGAATTTACCGTTCCAGTAAGCTTTTTTTTT
165	TGAATCGATTATATGTACCCCGGTTGATATTTTTTTTT
166	TCGTTGAATTAATAGAAAGGAACAACTAAAGGAATTTTTTTTTTT
167	TCGCACGATTGCCCGAGATAGGGTTGAGTTTTTTTTTT
168	TATCCGCTTTTAAAGTGTAAAGCCTGGGGTTTTTTTTT
169	TATAACCCTTCCAGCTATTGGGAGGTTTTGAAGCCTTAAATCAAGTTTTTTTTT

No.	Sequence (5’ to 3’)
170	TAGAGCAATTTAAAAACCAAAATAGCGAGTTTTTTTTTT
171	TACGAGGGTAGCAACGGCTATTTTTTTTTT
172	TAATCATATTGACGACAGTATCGGCCTCATTTTTTTTTT
173	TTTTTTTTTTGGTTGTGAATTCATGTTTGGATGTTCTTCTAAGTTTTTTTTTT
174	TTTTTTTTTTGCCTAATGAGTGAGCTAAT
175	TTTTTTTTTTCAATATCTGGTCAGTTTAATATCAAACCCTCAATTTTTTTTTT
176	TTTTTTTTTTAATTTTCCCTTAGAATCCTTATTTATCAAAATCATAGGTTTTTTTTTT
177	TTTTTTTTTTAACAAAGCTGCTCATTTATTACCCAAATCAACGTTTTTTTTTT
178	TTTTTTTTTTGTTGTTCCAGTTTGGAACAT
179	TTTTTTTTTTGTCATACATGGCTTTTGATTTCACCCTCAGAACCGCCACCTTTTTTTTTT
180	TTTTTTTTTTGTAGGGCTTAATTGAGTTAAGCCAACGCTCAACATTTTTTTTTT
181	TTTTTTTTTTGGAAGATCGCACTCCAGCCTTGCCAGGGT
182	TTTTTTTTTTGCGCATCGTAACCGTGCATTTGTCAACCT
183	TTTTTTTTTTGCGAATAATAATTTTTTCATTGCAGATAT
184	TTTTTTTTTTGCCTGAGTAGAAGAACTCATTAACAGGAAAAACGCTCATGTTTTTTTTTT
185	TTTTTTTTTTGCATTAACATCCAATATTTCTACTAATAGTAGTATTTTTTTTTT
186	TTTTTTTTTTGAAATACCTACATTTTGACGCTCAATTTCCATTGCTTAACTATCT
187	TTTTTTTTTTCTTTTGATAAGAGGTCATTTTCATCAATT
188	TTTTTTTTTTCTGAGAGACTACCTTTTTAACCTCCGTTATAGTGATTTTGAAAAT
189	TTTTTTTTTTCTCAGAACCGCCACCCTCATTCGTAACACTGAGTTTCGTCTTTTTTTTTT
190	TTTTTTTTTTCTAGGGCGCTGGCAAGTGTTTATTAGTAATAACATCACTTTTTTTTTTT
191	TTTTTTTTTTCGCATAGGCTGGCTGATTCGGTGTACAGACCAGGTTTTTTTTTT
192	TTTTTTTTTTCCTGTGTGAAATTGTTTATGGTCATAGCTGTTTTTTTTTTTT
193	TTTTTTTTTTCCGTCACCGACTTGAGTTTAAAGGTGAATTATCATTTTTTTTTT
194	TTTTTTTTTTCCGAAATCGGCAAAATCCCTTATAAATTGGCGAAATTTATTTACT
195	TTTTTTTTTTCCAGAAGGAAACCGAGGAAACGCAATTTAGTAAGCTTCAATGAAT
196	TTTTTTTTTTCCAAAATAACCCCGCTTTTTATGACAATGTCCCGTTTTTTTTTT
197	TTTTTTTTTTCATCAATATAATCCTGTTTCAGATGATGGCAATTTTTTTTTTT
198	TTTTTTTTTTCAGTGCCACGCTGAGATTTTAACACCGCCTGCAATTTTTTTTTT
199	TTTTTTTTTTCAGAGGCTTTGAGGACTAATTGATATTCT
200	TTTTTTTTTTCAATAATAAGAGCAAGAAATTAGATAGCCGAACAAAGTTATTTTTTTTTT
201	TTTTTTTTTTATTAGTTGCTATTTTGCACTTACAAGAATTGAGTTAAGCCTTTTTTTTTT
202	TTTTTTTTTTATTACGAGGCATAGTATTCATAACGCCAAAAGGATTTTTTTTTT
203	TTTTTTTTTTATCAGAAAAGCCCCAAAAATTCGTAATCT

No.	Sequence (5' to 3')
204	TTTTTTTTTTATAAAGCTAAATCGGTTTAATAAAGCCTCAGAGCTTTTTTTTTT
205	TTTTTTTTTTAGGTCAGACGATTGGCTTGACAGGAGGTTGAGGCTTTTTTTTTT
206	TTTTTTTTTTAGGCTTTTGCAAAAGAAGTTTGCTATCAT
207	TTTTTTTTTTAGAGAATATAAAGTACTTTTTCGAGCCAGTAATATTTTTTTTTTT
208	TTTTTTTTTTACGTCACCAATGAAACTTATTAGCAAGGCCGGAATTTTTTTTTTT
209	TTTTTTTTTTACCAGTACAAACTACAACGCCTGTAGTTCATGTACTTGAGCCACT
210	TTTTTTTTTTACCAGACCGGAAGCAAACTTTATTAAGCT
211	TTTTTTTTTTAATACGTAATGCCACTACGTTAGATGAAT
212	TTTTTTTTTTAAGAAATTGCGTAGATTTTTTGTAAATCGTCGCTATTAATTTTTTTTTTT
213	TTTTTTTTTTAAATCAGATATAGAAGTTGCGCCCAATAGCAAGCTTTTTTTTTT

Supplementary Table 2 | Sequences of staple strands of T1M.

No.	Sequence (5' to 3')
1	tTATTTTGCACCTTCATCAAGAGTAttGCGCATAGGCTGGCTGt
2	tCTTGCGGGAGGTTTTGAAGCCTTAttAAAAGCCTttAAAACAGGGAAGCGGt
3	tAGAACGCGAGGCGTTTTAGCGAACttGGCAGAGGt
4	tTCACCCAAGAGAACAAGCAAGCCGttAAGTACCGCACTCATCt
5	tAAAAACCGTCTATCAGGGCGATGGttCCGAGCTCttCCACCACACCCGATCt
6	tTAAAGAACGTGGACTCCAACGTCAttAATGAGTGt
7	tCCTTATGCGGTGGTTCCGAAATCGttGAAAATCCTGTTTGATt
8	tCTTGAGATGGTTTAATTTCAACTTttCGAAAGACttTAGTAAGAGCAAGACt
9	tCGAGAAACACCAGAACGAGTAGTAttCACCAACCt
10	tGTAAAGTAATTCTGTCttTTACCGCGttCAATCCAACATTCCAAt
11	tACCCCCAGCGATTATAttTTACCCAAttATTCATCAACAGATGAt
12	tGTCGGGAAACCTGTCGttAATAGCCCttGAGAAAGGCCACGCTGt
13	tGCAGACGGTCAATCATttTCCGCGACttATAATCAGttAAATATTTttCTCAGAGt
14	tAAAATAGCttAAAGTCAGAGGAGAAAACTTTTTCt
15	tCAAAAATGttCATTTTCGttTTTGAAATACCGACCGttGCTTAGGTttATTGAGCGt
16	tATTTACGAGCATGTAGttAGATAAGTttTATAAACAttATTCTGAAttCACCGGAt
17	tGCTGGCAAttGTTGCTTTGACTGCACTCTGTGGTt
18	tGCTAGGGCttAGCTAACTttGGTTTCTGCCAGCACGttTACCTGCAttCACGTATAt
19	tGCTGATTGCCCTTCACttCAGGGTGGttAGATGATGttTTATACTTt
20	tAGATACATttAACCAAAATAGACAGCTTGATACCt
21	tACTAATGCttTAAAACGAttCGATATATTCGGTCGCttAAAAGGCTttGAGGCTTTt
22	tTGGCTTTTttAACGGGGTttAGGTTGAGttACCCTCAGAACCGCCAt
23	tAGTAACATttGGAGCGGAttTTAGAGCCttAGGAAGGTTATCTAAAt
24	tGTCTGGAGttAACTAGCAttGACAGTCAttTGTAATACTTTTGAGGt
25	tTTTTGTCAttGGATATCAGGTCATTGCCTGAGAttACCCAGCTt
26	tTAAGTTTTttGATGATACttAAAGCCAGAATGGAAAGCGCAGTTttTGACAGGt
27	tCAGTGCCTTGAGTACttCCATCCTttATTTTGGttGGGTGAACCAt
28	tCTACATTTttTTTGTTCCAGTAAGCGTCATACAttATCAAGTTt
29	tCACCAGAAttTATCATTTttAATTCGACAACTCGTATTAAATCCttTAATAGAt
30	tATTATCATCATAGTCttGGGCAACttAGAACTGttGCGTGAATTAt
31	tAGGATTAGttACATTATTAATTTTAAAAGTTTGttGATTTTAAt
32	tAATCGTAAttCAAACAAGttGGAGAGGGTAGCTATTTTTGAGAAttGGCCGGAt
33	tTGTCAATCATATTTAttAACGAGGttCACAATTttTTTTAGTTGCt

No.	Sequence (5’ to 3’)
34	tAGTGTACCCCGGTTGttCTGCTCCAttATTTTCAGGGATAGCAt
35	tGCTCATGGttAGCACCAGCAGAAGATAAAACAGAGGTGACAAATCAACAt
36	tCAGTTCCTGATTATCttTTTTTCTTttCGTGGTGCTGGTCTGGt
37	tAACTCCAAttAGGTGTTTAGCTATATTTTCATTTGGGGCGTTGTACCAAt
38	tAATACCCAttACGGAATAttCAAGATAGCAGCACCGt
39	tCTTATTACttGTGGCAACATATAAAAttCATATGGTttCAGAGAGAttCGAGCGTCt
40	tGTTAGCATATTTCGGttGAAACAAAttCCTGTTTATCAACAATttAAACCAATt
41	tAGTAGAAGttATATTACCGCCAGCCAttGAAATGGAttGTCACGCTttTCGGAACCt
42	tACTATCGATCCTGATttAACCGCAAttTTTGCGTATTGGGCGCttCGCCTGGCt
43	tAAAAGATTttTTCGAGCTTCAAAGCGttCTCCTTTTttAAGGAATTttGGAAGAAAt
44	tAGCCCGAGGAAGATTttAGGAACCCttATAAATTGTGTCGAAAttAAGGGAACt
45	tGCCGAACAAAGTTACCAGttTAAGACTCttAGAGAGATttACTGAACAt
46	tCCGAGTAAAAGAGTCTGTttCTTGCCTGttTCCTCGTTttCAGGGCGCt
47	tAAAATCAGttTTCATCAAttCGTCTGGCCTTCCTGTAATTGCGTt
48	tCAGAAAACGAGAATGACCttTTGCATCAttAAGTTTTGttACCAGACGt
49	tAATAAAGAttCGTGGGAAttTGCCGGACTTGATGTTTAGACTGt
50	tTGATATAAGTATAGCCAACCAATAttCAAAGAATttTAATTAATATTTTGTt
51	tTGAATATACAGTAACAGTACCAGGttTAGCGTTTttGCATAAGAGGCTGAGt
52	tTCATAGCCCCCTTATttCGGATAAGttGCGGGGTTTTGCTCAt
53	tCACCTTGCttCCTAGGGTTAGAACCAttCGGAACGCttAAACGGCt
54	tATCATACAGGCAAGGttGGAACGCCttATCAGCTCATTTTTTt
55	tTTTAATGGttAGCCAGTAATAAGAGAttGTATTCTAttAACAGCCATAt
56	tATACCGGGttCACATTAATTGCGTTGttTCCACTATttGGAAAGCCGGt
57	tCGCATAACttAAGAGGCAAAAGAATAttTGCCCTGAttACATTATTACt
58	tAATAAAGCttAAATTGTAAACGCGGCttCACCCTCt
59	tGCCTTTAGCGTCACAAttAGGATAAAAATTTTTAGAACCCTt
60	tTCAAGTTTttGGCATGATttAAGGAAACttAGAGCAAGttTTTAGTACttACGTTGTt
61	tATCAAAATttACATGAAAGTATATATttCATTTCAt
62	tAACACCGCCTGCAATCttAGCCGCCACCAGt
63	tTCAGTATTttTAACATCAttCCATCACGttTTAAAGGGttGATTCGCCttGTTGTGTt
64	tCAATCAAttAAAAATCTAAAGCATttAGATTTTCttAAACAGAt
65	tCAAACCCTttCTGAATAATGGAGACAttACGTCAGt
66	tGTGGCATCAATTCACCTCAAATATttATATCTTTt
67	tCTGAAAAGttAAAGCGGAttATAAATCAttGATAGCGTttGAGTAACAttAGTTTGAt

No.	Sequence (5' to 3')
68	tCCCTCAGttCCCCTGCCttACTGGTAAttCAATAATCttATTAAACCt
69	tGTTGAAAttTCAATATAttAAAGAAACttCCTGAGAGttCAGCAGGCt
70	tAAACATTttCAAAAACAttTGAACGGTttCGAACTGAttCAGACCAGt
71	tCAATGAAACCATCAAGGGTGAGAAttGATCTACAAAGGCATTAGAGCCAGt
72	tTACCGAACGAACCCGCCGCCAGCAttCTCTGAATTTACCTGAATGGCTATt
73	tATGGTCAATAACCAGCACTAACAAttCTTTGCCCGAACGGTTGATTCCCAt
74	tCAAAATCAttAACGTCACttAGAAAATTttGAAACGCAttGAGAATTAttAACCCACAt
75	tCCAGTAGCttACCGTCACCGACTTGAttAACGCTAAttATAACATAt
76	tTCCTCATTttAGGAGTGTGGGAGGGAttGAACGGGTttGGCTGTCTTTCCTTATt
77	tTAGTCTTTttATTAAAAAttAATCGTCTttTTGCAACAttCGCCGCTAttAGAATCAGt
78	tAATGCGCGttAAGAATACGTGGCACAttGCACTAAAttGCGCGTAAt
79	tTTACAAACttTGCGGAACAAAGGGACttGTTTGCCCttAGTTGCAGCAAGCGGTt
80	tATTCTGCGttTTTCGCAAttTTTAATTGttAACCAGACttCCTCGTTTttCCAGAGGGt
81	tAACGAGTAttTGTCTGGAAGTTTCATTCCATATAttAGAGTACCt
82	tTTAATGCCttAGAATCGAGAATATAAttACGGTGTAttCCAACTTTGAAAGAGGt
83	tTTTAAATGCAATGCCTGAGTAATGTGTCGGGAGAt
84	tCATATATttTAATCAGTAGCGAGGAACCGCCTt
85	tAACCACCACCAGGCCACCAt
86	tCCCTCAGttTTTCATAttCCCTCAGAttAAATAACAGTGCCCGCttCTGAACAAttTTACCt
87	tAGCCTTTATTTCAACGCAttAATTAAGCttTAAAGATTt
88	tTGGGTTATttATGGAAACAGTACATAttCCAAGCTTTCAGAGGTGGAGCCGt
89	tGCCAGCGGttGAGGTGTCCAGCATCAttATCCCGTAAAAAAAGCCGCACAGt
90	tCCAAAAGGttCACAGACAGCCCTCATttTCGGCCTCAGGAAGATCGCACTCt
91	tAAAACGACGGCCAGTGttAATCAATAttAAATAGCAttCTAATATCt
92	tAAACTTAAttAAAGAGACGCAGAAACttGAAAGGATTAGGATTAttTGCCGTCt
93	tACATCGACATAAAAAAttGCGGGGTCttCAGGAACGttACGTGCTTt
94	tACGTTGGTttGGATTGACCGTAATGGttATATTATTTGCACGTAttAGGTTTAt
95	tGGGGACGACGACAGTAttAGTTAGCGttGCGGAATCttTGCAAAAGt
96	tGGCGATTAttTGGCGAAAGGGGGATGttAATAAATTTTTGTTAAttATCAAAAt
97	tCGCCACCCTCAGAACttCGGAATAGttCGCAATAAttTAGGACTGTAGCGCGTt
98	tCCATGTTTACATAGCTATCTTACAGGttAAACAATGt
99	tTCGGCGAAttCTCCGTGGTGAAGGGAttAAGATGATttAACCTATTttGTTAATGCt
100	tTGATTGCTTTGAATAttGTACCTTTttCCGTTGTAttGCCACAGTGCCACGCTt
101	tCGGCAAACGCGTACGCCAGAATCACGttATTTTAGAt

No.	Sequence (5' to 3')
102	tCGATGCTGttCTCCGGCCAGAGCACAttTCAGCAGCttTGTTTGGAttGCAATTCAt
103	tACCCGTCGGATTCTCttAGCCAGCTttGTCTTTACttTGATACTAATAGTAGTt
104	tCATTCGCCATGTCATAAATATTCAGCttCCAATACTt
105	tGGAAACCAttCGGTGCGGGCCTCTTCttAGCCCAATttGTATAAGCttAAAAGCCCt
106	tACGTCGCGCAGAGGCGAATACAGGAGGCCGAttCAAATTAttATACATCGGt
107	tTCCTCATAttTACCATATCAAAAATCAGTGAGGCCAt
108	tGCTATTACttTAAAATTCGCATTGCTTTAAACAGTTt
109	tGCCACCGCCACCCTCAGAGAGCCCAATAATAttCGAGGAAttAGTGTATCAt
110	tTGAGCAAAAGttTAGCTCTCttACTCCTCAAGAGAAAGTAAGCAGATAt
111	tTGTTACTTAGCCGCATTAGACGGttAAGACACCttAAAGAACTt
112	tAGAAAAATAATCCGCGCTTAATGttGGAAAAACttATTAGTAAt
113	tTTCACCAGTGACACTATCATAACttCGGAAGCAttTCAGAAGCt
114	tATATAAAGTAttttCGACAAAAGttTTCAttttCTTCTGACCTAAAt
115	tCGCTCACTGCttttCGCTTTCCAttCGGGttttCGTTTTCACGGTCt
116	tCACTAAAACAttttTCATCTTTGttGACAttttCAACCATCGCCCAt
117	tTTATTTATCCttCCCAATAGCAAGCAAATCAGATttttTAGAAGGCTTATCCGt
118	tAGGTAGAAAGttATCAACGTAACAAAGCTGCTCAttttTCAGTGAATAAGGCTt
119	tCGAACGTGGCttGAGATAGGGTTGAGTGTTGTTCttttAGTTTGGAACAAGAGt
120	tTTAAAGGTttGCCAAAGACAAAAGGGCttttACATTCAACCGATTGAt
121	tAGGTAAATATTGttttCGGAAATTATTCAttTGCCAGTTt
122	tTCTGACCTttATTGGCAGATTCACCAGttttCACACGACCAGTAATAt
123	tATTCTGGCCAACttttGAGATAGAACCCTttATTTAGAGt
124	tTGCAACTAttGTCATTTTTGCGGATGGttttTTAGAGCTTAATTGCTt
125	tTGCTGTAGCTCAttttCATGTTTTAAATAttACGAACTAt
126	tTTACCAGCttGAATTATCttACCATTACTATTCACAAttttCAAATAAAt
127	tTTATTTACttGAAAGCGTttAACTGATAATTTAGAAGttttATTAGACTt
128	tGATAAGAGttAAGTACGGttGATTTAGTACCGTTCTAttttCTGATAAAt
129	tAACGTAGAAAAttttACATACATAAAGttGCAGTATt
130	tGCCTTGCTGGTttttATATCCAGAACAttAACTCAAt
131	tAAGACTTCAAAttttATCGCGTTTTAAttAAGAGGAt
132	tGAGAGGGTttCCGTACTCAGGCGAAGCCCTTttttTTAAt
133	tACGTCAGAttGAGAAACAATACTGAGAAGTGttttTTTTt
134	tATAATTCGttCATTAAATGTGATTGAATCCCttttCTCAt
135	tTTTCATCGGCATTttttTCGGttACCAGAGCCttttCCACCCAGAAt

No.	Sequence (5' to 3')
136	tGAGAGCCAGCAGCttttAATGttTATCTGGTCttttGTTGGGGCGGt
137	tAGCATTAACATCCttttATAAttCATAAAGCTttttAATCGGCGAGt
138	tAGCCGCCttGCAGGTCAGACGATTGGCttttTTGACATTAGCAAGGCCGGAt
139	tGGAATTGttGTCAATAGATAATACATTttttGAGGGCCCTAAAACATCGCCt
140	tATGACCCttAATCACCATCAATATGATttttTTCATTGACCATTAGATACAt
141	tATGTAAATttttCTGATGCAAATCCAAttTGTTATTTTAGTTAATt
142	tCCCCCTGCttttTCAGACGATCCAGCGttGCCGGCCAGAATGCGGt
143	tATTGTATCttttGTTTATCAGCTTGCTttCGGTTGCGCCGACAATt
144	tCCACGGGttATTTCATTTGAttttTTACCTTTTTTAttATAACTATt
145	tCAGCCAGttCAAACTACAACttttCCTGTAGCATTCttAGCCTTTAt
146	tGCGGCCTttTCCCACGCAACttttAGCTTACGGCTGttTGCCGGTGt
147	tCAGTCCCGGAATTTGttttGAGAGATAGACTTTttACGTACAGCGt
148	tGGTCCGTTTTTTCGTttttTCGTCGCTGGCAGCttATTGCCGTTCt
149	tTCAGGCTGCGCAACTttttTTGGGAAGGGCGATttGGCAAAGCGCt
150	tAAATATCAttATAAGAAACGATttttTTTTGTTTAACGTttACAAAATAt
151	tGCTAATGCAGAACGCGttCATCAAGAAAACACAAGACttttAAGAACGCGGTAt
152	tGATAGAGAttGTTGAGATTTAGttttAATACCACATTCAttACGGAACAt
153	tGCTGCAACttAAGGGAAGAAAGttttGAAAGGAGCGGGCttCTTGACGGt
154	tGCGCGGGGAGAGGCGGttGAATGCCAACGGCTGTCACttttGCGCGCCTGGAGt
155	tTTTGTATCATCGCCTGttATGTACCGTAACAGAGGTGttttATTTCTTAACGAt
156	tATCTTGACAAGAACCGGATATTCAttCCAAGCGCGAA
157	CCATATTTAACAAttGTAATTTAttCTCCCGATttTTCCAGAGCCTAATTt
158	tTATGTGAGttCCTTTTTTttTTCCCTTGttTAGGGCTTAATTGAGAATCG
159	ACAAAGTACAAttTTCCTGAGTTT
160	GCTATTAATTAATttAACCTCCGttTGTGATAAttCCCTGAACttAGCCTTTAt
161	CGTCACCAGTAttCTTTCCGGCACCGCTTCTGGTGCCTGCTGCAAt
162	tAGAGACTAttTGACTGAAttGACGATAACCTTGCTTCTGTAAATCGTC
163	TAATTACTAGAttAATCAAGAttATCCTGAATCTTACCGttCCATTTGCttAATCAATt
164	tATAAGGCttTCAACATttCGCCATATGCGTTATACAAATTCTTACCA
165	GTATAAAGCCAACttAGAATAAACACCGGAATCA
166	TATCAAAATCATAGGTCTGttAGAATTGAGTTACCAt
167	tGTTTAGTAttGTTAAATAttGCTCAACAttAGAATCCTTGAAAACATAGCG
168	tCGCCACCCTCAGAAGCttAGTTGGGTAACGCCAGGGTTTTCCttAATAGTGAATT
169	ATAGCTTAGATTAttGAAGAGTCttCAGTCACGt

No.	Sequence (5' to 3')
170	tTTTTTATTTTCATCGTAGGAATCAttCAGACGACGAC
171	GAAGCATAAAGTGttGGGTGCCTttAAGGGCGCttTAAAGGGAGCCCCCGt
172	tATTGCAGGttCATCAGAGttCCGGGTCTttTCCACACAACATACGAGCCG
173	AATAAACAACAttTCGAAATTAAT
174	GGTTGCGGTATGAttTGCCGGGTttCGTGCCTGttGTACTATGttGTGTAGCGt
175	TACATTTAACAttAACGGATAACCTCACCGGAAACAAAGCGGATCt
176	tGTTAACGGttCGCGCTCTttCTTTTTTCGCACTCAATCCGCCGGGCGC
177	ATCCCCGGGTAttCCCACTACttTCGAGGTGCCGTAAAGttACAATATTttGACGCTCt
178	tTTCTTCGttAATCCTGttTAAAGCATGGTCATAGCTGTTTCCTGTGT
179	GAAATTGTTATCCttAGCCTCCTCACAGTTGAGG
180	CTGGTGTGTTCAGCAAATCttAGCGGGAGCTAATATCttTTCTTTGt
181	tGAATTCGTttCGTCCGTGttGCTCACAAttACTGTTGCCCTGCGGCTGGTA
182	tCCAAGTTACAAAAGAAttATTTCTGCTCATTTGCCGCCAGCAttCATCCCTTACA
183	ATGGGTAAAGGTTttGTCATAAAttGTTGGGCGt
184	tGCAAAATCCCTTATAAATCAAAAGttTGCCAGCTGCA
185	CGGGTAAAATACGttTACGAAGGttAATTGGGAttATCTACGTTAATAAAt
186	tTAACGATCttTGAAAATTttCTGTATGCttATGAGGAAGTTTCCATTAAA
187	TTAATGAATCGttCAGAGCACCGT
188	AGTAAATGAATTTttCTCCAAAAttTGAGGCTTttACGATAAAttAACGCCAAt
189	CGGTGGTGCCAttTTAGTGATGAAGGGTAAAGTTAAAGATAGGTCt
190	tTTTCACGTttTAAAGAAGttAGTGAGTTTTGTCGTCTTTCCAGACGTT
191	ACCCTCAGCAGttTAATCATTttTCATTATACCAGTCAGGACGTTGAttCGAGGCAt
192	tGCAGGGAttAACCCACttTAATGGAGGGTAGCAACGGCTACAGAGGC
193	TTTGAGGACTAAAttCCGCTTTTGCGGGATCGTC
194	GAATTGCGAATAATAATTTttGGTAATAGTAAATAGTttATTATAGt
195	tAGCATCGGttGTTAAAGGttGACTTTTTttGGATTTTGCTAAACAACTTTC
196	tGTAGATGGGCGCATCGTAACCGTGttAACAACTAAAG
197	AACAGTTTCAGCGttTAGAAAGGttCATCTGCCt

Supplementary Table 3 | Sequences of modified staple strands of 42hb.

Name	Sequence (5' to 3')
192-5Cy5	Cy5_cctgtgtgaaattgttttatggtcatagctgttttttttttt
193-5Cy5	Cy5_ccgtcaccgacttgagtttaaaggtgaattatcatttttttttt
198-5Cy5	Cy5_cagtgccacgctgagattttaacaccgcctgcaattttttttt
202-5Cy5	Cy5_attacgaggcatagtattcataacgccaaaaggattttttttt
5-si	**gtgctactccagttcttt**tttttaactttgtatcaccgtactcaggttccgtaatcat
7-si	**gtgctactccagttcttt**ttttcagcggagtgagttaatctccttaagacagttttagactcct
18-si	**gtgctactccagttcttt**tttaaaggccttgtatcggtttatcagcttttacaacattat
20-si	**gtgctactccagttcttt**ttgctgaatttttcatttggggcgcgttagcatgtat
23-si	**gtgctactccagttcttt**ttgagaagtgttttattttgcccttttactcgtcttcagggctttaaatttt
25-si	**gtgctactccagttcttt**ttctgtatgggattttgctttttaattttgcttttgt
27-si	**gtgctactccagttcttt**ttcgtctttccagacgtttaggtgaattcggtcgcttccgtcgat
31-si	**gtgctactccagttcttt**ttcaggctgcttgccagggttttcccagtcttgaacgttatt
32-si	**gtgctactccagttcttt**ttcactgcccgctttccattctgaaagt
38-si	**gtgctactccagttcttt**ttataactatatgtaaatttaacgccat
50-si	**gtgctactccagttcttt**ttgtggcgagaaaggattacgctgcttcatattctttcaacagttataacctttcatagcgt
57-si	tgggcgcagaacaatattaccgccagttcgtctgatt**tttcttgacctcatcgtg**
59-si	**gtgctactccagttcttt**ttggcaaaattggaaccgaactgaccaattatcaccggt
68-si	**gtgctactccagttcttt**ttgcggggagaggcggtttttcaccagtt
72-si	**gtgctactccagttcttt**ttgcccgtattgagggttgatataagtatttttagcgtt
79-si	**gtgctactccagttcttt**ttgacccccattgccggaacgaggcgcagattcctccctcat
90-si	**gtgctactccagttcttt**ttctcacatttggaagcattcacaattccacacattaccgagcttgctcgcct
94-si	**gtgctactccagttcttt**ttctaaataactagcatttattgtatttccgtaatttttgagcaaaagaagat
104-si	**gtgctactccagttcttt**ttccggaaattcgacggccagtgccaagttaattcgact
113-si	**gtgctactccagttcttt**ttcataaaggtggcaacatttagcgtcttaaaaagattagaataat
120-si	**gtgctactccagttcttt**ttcaactaaatcggaatttgctttgttaagtttgttagattagttaacaattttaaatgtgt
123-si	**gtgctactccagttcttt**ttatttagagcttgatttaatgcgttaaagaaatttaaaatattgaaacagt
124-si	**gtgctactccagttcttt**ttatttacattggcagattttgcgtattt
129-si	**gtgctactccagttcttt**ttatcgcaagacaaagaatttgttaaattcaacatttttcatttgt
132-si	**gtgctactccagttcttt**ttagtttcatttcgagtagatttagtttgttccaagtacct
134-si	**gtgctactccagttcttt**ttagtaataaaagggacttgaatcggttagctgatt
138-si	**gtgctactccagttcttt**ttaggagaattagctttcttaggggacttcaggcaaggcaacgt
142-si	**gtgctactccagttcttt**ttagagtccttaagaatattcttaagtgtcctacttctttctct
145-si	**gtgctactccagttcttt**ttacctcccttctaagaacgcgaggcgttttccgtttttat

Name	Sequence (5' to 3')
148-si	**gtgctactccagttcttt**ttactccaacgtcaaattcgttagatttaaatccttggatttattcaaacatt
163-si	**gtgctactccagttcttt**tgtttccattaaacgggtaattttttttt
171-si	**gtgctactccagttcttt**ttacgagggtagcaacggctattttttttt

Supplementary Table 4 | Sequences of siRNA.

Name	Sequence (5' to 3')
luci-si-Passenger-capture	**gaacuggaguagcacaaa**gauugacaaauacgauuuauc
luci-si-Guide	uaaaucguauuugucaaucag
